# Coordinatively Unsaturated Nickel Nitroxyl Complex: Structure, Physicochemical Properties, and Reactivity toward Dioxygen

**DOI:** 10.3390/molecules28176206

**Published:** 2023-08-23

**Authors:** Kiyoshi Fujisawa, Taisei Kataoka, Kohei Terashima, Haruka Kurihara, Felipe de Santis Gonçalves, Nicolai Lehnert

**Affiliations:** 1 Department of Chemistry, Ibaraki University, Mito 310-8512, Ibaraki, Japan; 2 Department of Chemistry and Department of Biophysics, University of Michigan, 930 N. University Avenue, Ann Arbor, MI 48109, USA; felipe.sg@usp.br

**Keywords:** nickel, nitrosyl, crystal structure, oxidation reaction, noninnocent ligand, N2 ligand

## Abstract

For its important roles in biology, nitrogen monoxide (·NO) has become one of the most studied and fascinating molecules in chemistry. ·NO itself acts as a “noninnocent” or “redox active” ligand to transition metal ions to give metal–NO (M–NO) complexes. Because of this uncertainty due to redox chemistry, the real description of the electronic structure of the M–NO unit requires extensive spectroscopic and theoretical studies. We previously reported the Ni–NO complex with a hindered N3 type ligand [Ni(NO)(L3)] (L3^−^ denotes hydrotris(3-tertiary butyl-5-isopropyl-1-pyrazolyl)borate anion), which contains a high-spin (hs) nickel(II) center and a coordinated ^3^NO^−^. This complex is very stable toward dioxygen due to steric protection of the nickel(II) center. Here, we report the dioxygen reactivity of a new Ni–NO complex, **[Ni(NO)(I)(L1″)]**, with a less hindered N2 type bis(pyrazolyl)methane ligand, which creates a coordinatively unsaturated ligand environment about the nickel center. Here, L1″ denotes bis(3,5-diisopropyl-1-pyrazolyl)methane. This complex is also described as a hs-nickel(II) center with a bound ^3^NO^−^, based on spectroscopic and theoretical studies. Unexpectedly, the reaction of **[Ni(NO)(I)(L1″)]** with O_2_ yielded **[Ni(κ^2^-O_2_N)(L1″)_2_](I_3_)**, with the oxidation of both ^3^NO^−^ and the I^−^ ion to yield NO_2_^−^ and I_3_^−^. Both complexes were characterized by X-ray crystallography, IR, and UV–Vis spectroscopy and theoretical calculations.

## 1. Introduction

Nitrogen monoxide (·NO) is a small, relatively unstable, potentially toxic, diatomic free-radical molecule. ·NO has become one of the most studied and fascinating molecules in biological as well as inorganic chemistry [1,2,3,4,5,6,7,8,9,10]. Nitrogen monoxide itself acts as a ligand to transition metal ions to give metal–NO (M–NO) complexes [1,5,6,7,8,9,10]. These M–NO complexes have been studied for a long time, due to the character of ·NO being one of the so-called “noninnocent ligands”, that is to say, a “redox active ligand” [11,12,13]. ·NO can change its oxidation state from ·NO (nitrosyl, *S*_NO_ = 1/2) to ^1^NO^+^ (by oxidation of ·NO, nitrosyl cation, *S*_NO_ = 0), and to NO^−^ (by reduction of ·NO, nitroxyl, *S*_NO_ = 0, 1) when binding to a transition metal ion, i.e., M^n+^–NO·, M^n−1^–NO^+^, and M^n+1^–NO^−^ [5,6,7,8,9,10,12,13]. By combination of recent powerful spectroscopic techniques and in-depth theoretical calculations, the correct description of the oxidation state of the [MNO] unit can be obtained. The famous notation, which was introduced by Professors Enemark and Feltham in 1974, is still used to describe these difficult oxidation states [14]. In the Enemark–Feltham notation, {M(NO)*_x_*}*^n^*, the M(NO)*_x_* entity (*x* = number of bound NO ligand(s)) is treated as a covalent unit where the superscript (index) *n* denotes the number of valence electrons = metal (d) + NO (π*) electrons of the [M(NO)*_x_*] complex.

**Figure 1:**
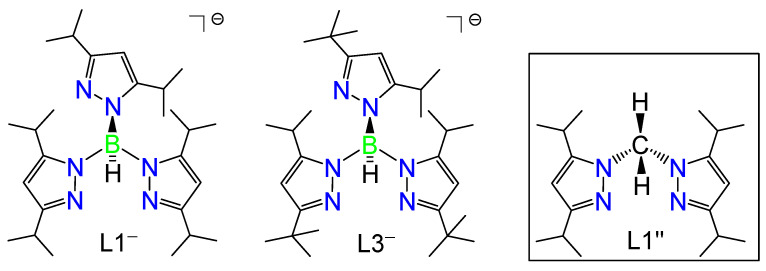
Structures of the ligands previously utilized by our groups: hydrotris(3,5-diisopropyl-1-pyrazolyl)borate (L1^−^), hydrotris(3-tertiary butyl-5-isopropyl-1-pyrazolyl)borate anion (L3^−^), and the ligand bis(3,5-diisopropyl-1-pyrazolyl)methane (L1″), used in this work.

We are interested in the coordination structures of transition metal ions, and how these affect their spectroscopic properties as well as their reactivity toward small molecules. We previously reported the syntheses of metal(II) thiolato complexes by using the first-row transition metals Mn(II), Fe(II), Co(II), Ni(II), Cu(II), and Zn(II) to obtain more insight into the structural and spectroscopic properties of blue copper proteins, by studying the analogs [M(SC_6_F_5_)(L1)] [15,16]. This research is also aimed at understanding the relative rigidity of the hydrotris(pyrazolyl)borate coligand, hydrotris(3,5-diisopropyl-1-pyrazolyl)borate ligand (denotes as L1^−^ in Figure 1, left [17]), and its ability to accommodate the first-row transition metal(II) ions [15,16]. We have observed subtle angular and torsional variations within this ligand framework to accommodate the ionic radii of the first-row transition metal(II) ions and their general coordination structure preference, according to the Irving–Williams series [18]. Later, we also explored the first-row M–NO complexes with a more hindered hydrotris(pyrazolyl)borate coligand, hydrotris(3-tertiary butyl-5-isopropyl-1-pyrazolyl)borate anion (denoted as L3^−^ in Figure 1, center [19]), to control the coordination numbers of the complexes and keeping them four-coordinate, with a distorted tetrahedral geometry, in the high-spin (hs) state. We obtained a series of four-coordinate hs-{MNO}^8−11^ complexes [7,10] with M = Fe [20], Co [21], Ni [22], and Cu [23]. Using detailed spectroscopic investigations coupled to DFT calculations, we were able to show that their electronic structures are best described as M(II)–NO^−^ complexes for Fe, Co, and Ni, where the corresponding hs-M(II) center is coordinated to a ^3^NO^−^ ligand. These complexes are of hs-{FeNO}^8^, hs-{CoNO}^9^, and hs-{NiNO}^10^ type, respectively [7,10,20,21,22]. This is in stark contrast to the {CuNO}^11^ analogue, which contains a coordinated ^2^NO· ligand [7,10,23].

In our previous research, the corresponding Ni(II)–NO complex [Ni(NO)(L3)], an hs-{NiNO}^10^ system, did not show any reactivity toward dioxygen [22]. This is caused by the protection provided by the hindered tertiary butyl groups at the nickel center. Therefore, we used bis(pyrazolyl)methane as an N2 type ligand in this work, removing one pyrazole ring with a less hindered isopropyl group to reduce steric hindrance at the metal center, creating an accordingly unsaturated ligand environment. In this way, small molecules—such as dioxygen—can interact better with the nickel center. We used the ligand bis(3,5-diisopropyl-1-pyrazolyl)methane (=L1″) [24] (Figure 1, right). We previously investigated the coordination chemistry of this ligand in copper(II) nitrito complexes with L3^−^ and L1″, [Cu(κ^2^-ONO)(L3)] and [Cu(κ^1^-ONO)_2_(L1″)]. The main differences between these complexes relate to the different ligand charges (an anion versus a neutral species) and bulkiness (tertiary butyl versus isopropyl substituents) [24]. We also prepared the analogous copper(II) chlorido complexes with L3^−^ and L1″, [Cu(Cl)(L3)] and [CuCl_2_(L1″)], which again differ for the same reasons mentioned above [25,26]. This work expands the literature precedents of four-coordinate Ni–NO complexes with hydrotris(pyrazolyl)borate analogues, which are listed in Table 1: [Ni(NO)(L3)] [22], [Ni(NO)(L0f)] [27] (L0f^−^ = hydrotris(3-trifluoromethyl-5-methyl-1-pyrazolyl)borate anion), [Ni(NO)(L0)] [28,29] (L0^−^ = hydrotris(3,5-dimethyl-1-pyrazolyl)borate anion), [Ni(NO)(iPr_3_tacn)](PF_6_) [30] (iPr_3_tacn = 1,4,7-triisopropyl-1,4,7-triazacyclononane), [Ni(NO)(Tm*^p^*^-tol^)] [31] (Tm*^p^*^-tol−^ = hydrotris(2-mercapoto-3-*p*-tolyl-1-imidazolyl)borate anion), [Ni(NO)(Tse^Mes^)] [28] (Tse^Mes−^ = hydrotris(2-seleno-3-mesityl-1-imidazolyl)borate anion), [Ni(NO){HB(*^t^*BuIm)_3_}] [32] (HB(*^t^*BuIm)_3_^−^ = hydrotris(3-tertiary butyl-imidazolyl-2-ylidene)borate anion), [Ni(NO){PhB(CyCH_2_Im)_3_}] [33] (PhB(CyCH_2_Im)_3_^−^ = phenyltris(3-cyclohexylmethyl-imidazolyl-2-ylidene)borate anion), [Ni(NO){HB(MeBz)_3_}] [33] (HB(MeBz)_3_^−^ = hydrotris(3-methyl-imidazolyl-2-ylidene)borate anion), and [Ni(NO){HB(*p*-^t^BuPhTz)_3_}] [33] (HB(*p*-^t^BuPhTz)_3_^−^ = hydrotris(3-*p*-tertiary butyl-phenyl-imidazolyl-2-ylidene)borate anion).

Compared to [Ni(NO)(L3)], [Ni(NO)(L0f)] is also stable toward dioxygen due to the hindered CF_3_ group [27]. In this research, we explored the dioxygen reactivity of the Ni–NO complex **[Ni(NO)(I)(L1″)]** with a less hindered N2 type bis(pyrazolyl)methane coligand to create a coordinatively unsaturated ligand environment. We obtained an unexpected, six-coordinate compound, **[Ni(κ^2^-O_2_N)(L1″)_2_](I_3_)**, with the oxidation of ^3^NO^−^ and the I^−^ ion by O_2_ to yield NO_2_^−^ and I_3_^−^ as the product, with some rearrangement of the L1″ coligand as well. Both obtained complexes were characterized by X-ray crystallography, IR, and UV–Vis spectroscopy and theoretical calculations.

## 2. Results and Discussion

### 2.1. Synthesis of Ni–NO Complex

**Figure 2:**
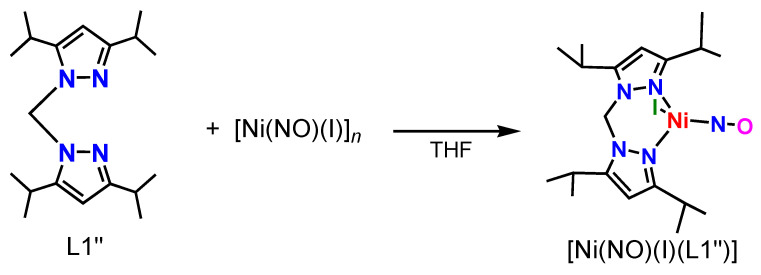
Synthesis of the nickel(II) nitrosyl complex **[Ni(NO)(I)(L1″)]**.

The first structural characterization of a Ni–NO complex [Ni(NO)(N_3_)(PPh_3_)_2_] was reported in 1971 by Professor Enemark [34]. Later, many nickel–nitrosyl structures were reported for inorganic salts and complexes coordinated by a variety of auxiliary coligands. The first {NiNO}^10^ complex with an N3 type hydrotris(pyrazolyl)borate coligand, [Ni(NO)(L0)], was obtained using [Ni(NO)(Br)(PPh_3_)_2_] as the precursor [28]. The Ni–NO complex [Ni(NO)(L3)] was obtained by the reaction of the abovementioned starting material [Ni(NO)(Br)(PPh_3_)_2_] with the potassium salt of the ligand [22]. However, we thought that simple nickel–NO complexes such as [Ni(NO)(I)]*_n_* could also be used for the synthesis [35]. In fact, [Fe(NO)_2_(I)]_2_ and [Co(NO)_2_(I)]*_n_* were used to synthesize M–NO complexes such as [Fe(NO)(L3)] [20], [Fe(NO)_2_(L1″)](BF_4_), and [Co(NO)_2_(L1″)](BF_4_) [36] previously. Moreover, we succeeded in the synthesis of [Ni(NO)(L0f)] by using both [Ni(NO)(Br)(PPh_3_)_2_] and [Ni(NO)(I)]*_n_* [27]. Therefore, we selected [Ni(NO)(I)]*_n_* as starting material in this research. The Ni–NO complex **[Ni(NO)(I)(L1″)]** was obtained by the reaction of [Ni(NO)(I)]*_n_* and the ligand L1″, as shown in Figure 2. After the reaction and the subsequent slow recrystallization at −30 °C, we obtained green crystals of **[Ni(NO)(I)(L1″)]**, suitable for structural characterization. The coordinated iodide ion is needed for the structural stability of the complex. If we add a THF solution of AgPF_6_ to a solution of **[Ni(NO)(I)(L1″)]** to remove the iodide ion, the color of the solution changes from green to colorless, indicating that, upon removing the iodide, the complex decomposes.

### 2.2. Structure of Ni-NO Complex

**Figure 3:**
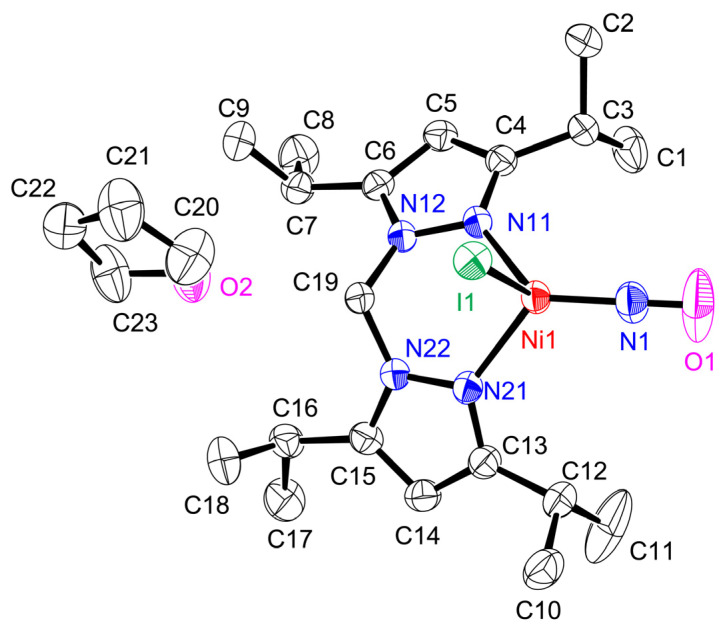
Molecular structure of **[Ni(NO)(I)(L1″)]**(thf) showing 50% thermal ellipsoids and the atom-labeling scheme. Hydrogen atoms are omitted for clarity. Relevant bond lengths (Å) and angles (°). Ni1–N1, 1.6467 (16); N1–O1, 1.136 (3); Ni1–N11, 2.0157 (12); Ni1–N21, 2.0234 (12); Ni1–I1, 2.6297 (4); Ni1–N41–O41, 176.68 (19); N11–Ni1–N1, 123.86 (6); N21–Ni1–N1, 102.04 (4); N11–Ni1–N12, 91.41 (5); N1–Ni1–I1, 112.02 (6); N11–Ni1–I1, 99.64 (4); N21–Ni1–I1, 91.36 (12).

The structure of **[Ni(NO)(I)(L1″)]** is shown in Figure 3, with the relevant bond lengths (Å) and angles (°) listed in the figure caption. One tetrahydrofuran molecule exists as a solvate with some interaction between its oxygen atom and the C1′ atom (3.413 (3) Å) and C3′ atom (3.480 (3) Å) to stabilize the crystals (symmetry operator ‘: −X + 3, −Y + 1, −Z + 1).

The coordination geometry of Ni in **[Ni(NO)(I)(L1″)]** is four-coordinate with three nitrogen atoms from L1″ and NO and the iodide bound. The Ni1–N11 and Ni1–N21 bond lengths (2.0157 (12) and 2.0234 (12) Å) are experimentally equivalent and considerably longer than the Ni1–N1 (1.6467(16) Å) bond. The N1–O1 bond length is 1.136 (3) Å and the Ni1–N1–O1 angle is 172.42(17)°. The presence of the bidentate ligand, with a restricted bite angle, introduces significant distortions in this coordination geometry. Thus, the variation in the N–Ni1–N angles is substantial, with the narrowest angle being 91.41 (5)° for N11–Ni1–N21 and the widest being 123.86 (6)° for N1–Ni–N11. The value of *τ*_4_ = [360 – (*α* + *β*)/141] = 0.80, where *α* and *β* are the two widest angles subtended at the metal center [37]. The value compares with 0.00 for an ideal square-planar geometry, 0.85 for a trigonal-pyramidal arrangement, and 1.00 for a tetrahedral geometry. Indeed, the value of *τ*_4_ in **[Ni(NO)(I)(L1″)]** is closest to 0.85, which corresponds most closely to a trigonal pyramidal geometry (*C*_3v_). In this structure, the three nitrogen donors form the basal (trigonal) plane. To investigate this further, we calculated the distances between the basal planes formed by three of the coordinating atoms and the nickel center. Here, the distance between Ni1 and the plane formed by N11, N21, and I1 is much longer (1.0817 (3) Å) compared to the other three: 0.4900 (3) Å between Ni1 and the plane formed by N11, N21, and N1; 0.5673 (3) Å between Ni1 and the plane formed by N11, N1, and I1; and 0.5493 (3) Å between Ni1 and the plane formed by N21, N1, and I1. The latter three distances are quite similar. Based on these considerations, the coordination geometry of the complex is distorted tetrahedral, which is a different conclusion than the result from the *τ*_4_ value discussion.

The distances and angles are not so different compared to the values reported for other four-coordinate Ni–NO complexes, with some deviations, as listed in Table 1. The Ni–NO bond lengths range from 1.617 to 1.677 Å and the N–O bond lengths range from 1.123 to 1.197 Å, despite the usage of different types of ligand donor atoms in these compounds. Interestingly, however, the Ni–N–O bond angles range from 169.3° to 180°, and the ν(N–O) stretching frequencies range from 1693 to 1823 cm^−1^ in these compounds, showing a larger degree of variability and indicating distinct differences in the electronic structures of these complexes. Therefore, careful spectroscopic experiments as well as theoretical support are very important to describe the true electronic structure of **[Ni(NO)(I)(L1″)]**, as well as the oxidation state of the metal center and the NO group [10].

### 2.3. Characterizations of Ni-NO Complex

IR and far-IR spectra of **[Ni(NO)(I)(L1″)]** were measured using KBr and CsI pellets, respectively (Figures S1 and S2). The ν(N–O) value of this complex is 1777 cm^−1^ (Figure S1), which is clearly shifted from 1854 cm^−1^ observed in the starting material [Ni(NO)(I)]*_n_* (Figure S3). Considering the electron-donating properties of hydrotris(pyrazolyl)borate type ligands, the following order in ν(N–O) frequencies is observed for varying pyrazolyl substituents: 1823 cm^−1^ in [Ni(NO)(L0f)] [27] > 1780 cm^−1^ in [Ni(NO)(L3)] [22] > 1777 cm^−1^ in **[Ni(NO)(I)(L1″)]**, as listed in Table 1. The Ni–NO stretching vibration, ν(Ni–NO), is identified at 572 cm^−1^, and is assigned considering related values, 574 cm^−1^ in [Ni(NO)(L3)] [22] and 598 cm^−1^ in [Ni(NO)(L0f)] [27] (Figure S2). The direct comparison between L3^−^ and L1″ shows that, in spite of the different ligands (one nitrogen atom from pyrazolyl versus iodide coordination), the electronic structure of the Ni–NO unit in these complexes is very similar, reflected by their similar vibrational properties. On the other hand, a more electron-withdrawing substituent (as the CF_3_ groups in L0f^−^) can greatly influence the Ni–NO π interaction, as shown by the higher ν(N–O) frequency for [Ni(NO)(L0f)] [5,10].

**Figure 4:**
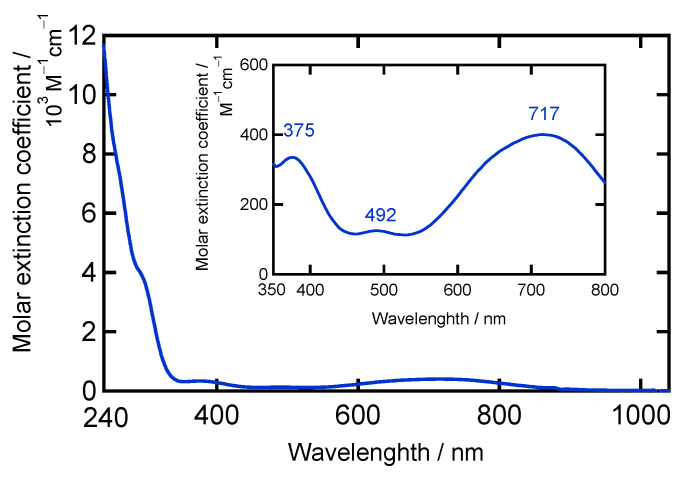
UV–Vis spectrum of **[Ni(NO)(I)(L1″)]** in CH_2_Cl_2_. Inset: expanding 350–800 nm region.

UV–Vis spectra (dichloromethane solution and solid state) of **[Ni(NO)(I)(L1″)]** are shown in Figure 4 and Figure S4. The similarities of the UV–Vis spectra in the solution and solid state indicate that the structure observed by X-ray crystallography is largely retained in solution. The observed absorption bands appearing in the 500–800 nm range are assigned to primarily Ni *d*–*d* transitions [22]. In [Ni(SC_6_F_5_)(L1)], the band at 804 nm is assigned to the ^3^T_1_(F) → ^3^T_1_(P) transition, giving rise to a pseudo A signal in MCD (magnetic circular dichroism) because of instate spin–orbit coupling [15,16]. **[Ni(NO)(I)(L1″)]** exhibits strong absorption bands in the 500–700 nm range, as shown in Figure 4. From the above assignments, these lower energy bands can be assigned to the spin–allowed *d*–*d* transitions of the high-spin Ni(II) center. Therefore, the UV–Vis absorption data indicate that the Ni oxidation number is +II, and that the Ni(II) center is in the high-spin state. This implies that the NO ligand is reduced in the complex to ^3^NO^−^ (nitroxyl), since the complex charge is zero. This is further discussed in the DFT section. The complex **[Ni(NO)(I)(L1″)]** has a green color {*λ*_max_/nm (*ε*/M^−1^cm^−1^): 492 (130) and 717 (400)}, whereas [Ni(NO)(L3)] {*λ*_max_/nm (*ε*/M^−1^cm^−1^): 630 (480)} [22] and [Ni(NO)(L0f)] {*λ*_max_/nm (*ε*/M^−1^cm^−1^): 593 (440)} [27] are blue. This difference would come from the different ligand donor set (**[Ni(NO)(I)(L1″)]** versus [Ni(NO)(L3)]), causing a weaker overall ligand field and a shift of the ^3^T_1_(F) → ^3^T_1_(P) type *d*–*d* transition to lower energy in **[Ni(NO)(I)(L1″)]** (717 nm) compared to [Ni(NO)(L3)] (630 nm) and [Ni(NO)(L0f)] (593 nm). These spectral features are different from those of the starting material [Ni(NO)(I)]*_n_* (Figure S3).

Nuclear magnetic resonance (NMR) spectra (^1^H and ^13^C) are compiled in Figures S5–S7. The Ni–NO complex **[Ni(NO)(I)(L1″)]** has relative sharp ^1^H-NMR signals in both CDCl_3_ and (CD_3_)_2_CO solution, as shown in Figures S5 and S6, indicating that the complex is diamagnetic. As mentioned above, the Ni oxidation state of **[Ni(NO)(I)(L1″)]** is +II, and the metal center is in the high-spin state. Therefore, the total spin of the Ni(II) center should be *S* = 1. However, the NMR spectra clearly indicate that the complex is diamagnetic (*S*_total_ = 0). This originates from antiferromagnetic coupling between the d-electrons of Ni(II) and the π*-electrons of the nitroxyl ligand (*S*_total_ = 0; open shell diamagnetic). This assignment was previously proposed for other complexes, listed in Table 1 [29,30,31,32,33]. The slight broadening of the NMR signals is likely related to a small amount of NO loss from the complex, forming a small fraction of a paramagnetic Ni(I) complex. Since no distinct signals associated with such a species are observed, this indicates that the amount of Ni(I) complex formed is small. This is in agreement with the UV–Vis absorption spectra.

### 2.4. Dioxygen Reactivity of Ni-NO Complex

**Figure 5:**
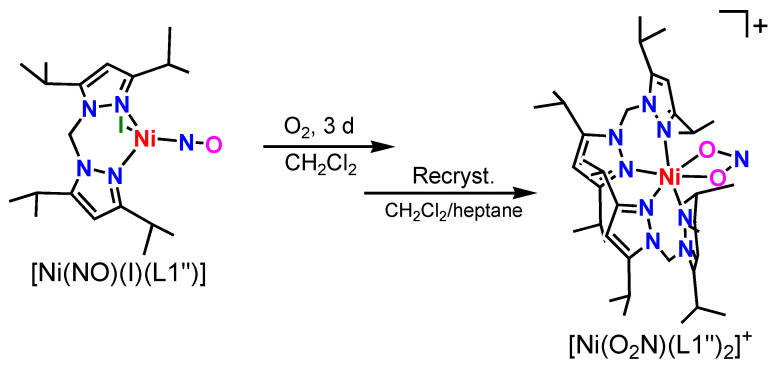
Dioxygen reaction of [Ni(NO)(I)(L1″)] to yield [Ni(κ^2^-O_2_N)(L1″)_2_](I_3_).

**Figure 6:**
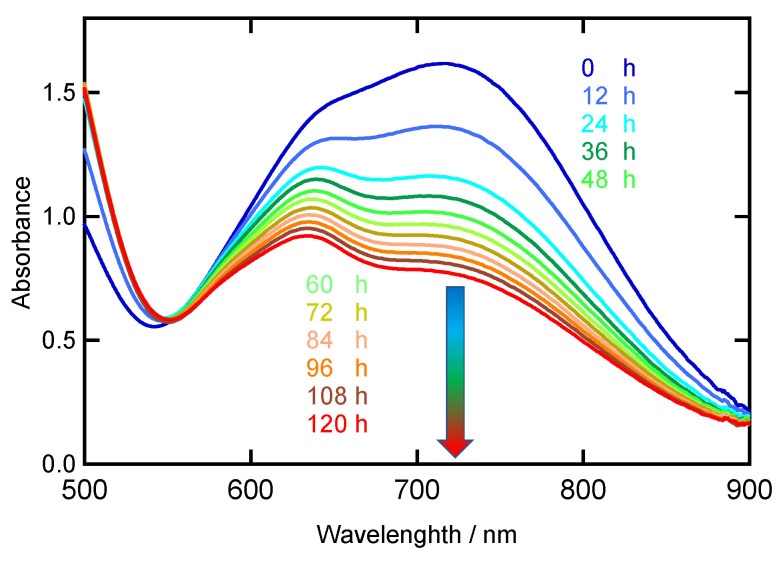
Time-dependent visible spectral changes, following the reaction of **[Ni(NO)(I)(L1″)]** with dioxygen in dichloromethane at room temperature, monitored every 12 h. The arrow schematically shows the progress of the reaction as a function of time (blue to red).

**Figure 7:**
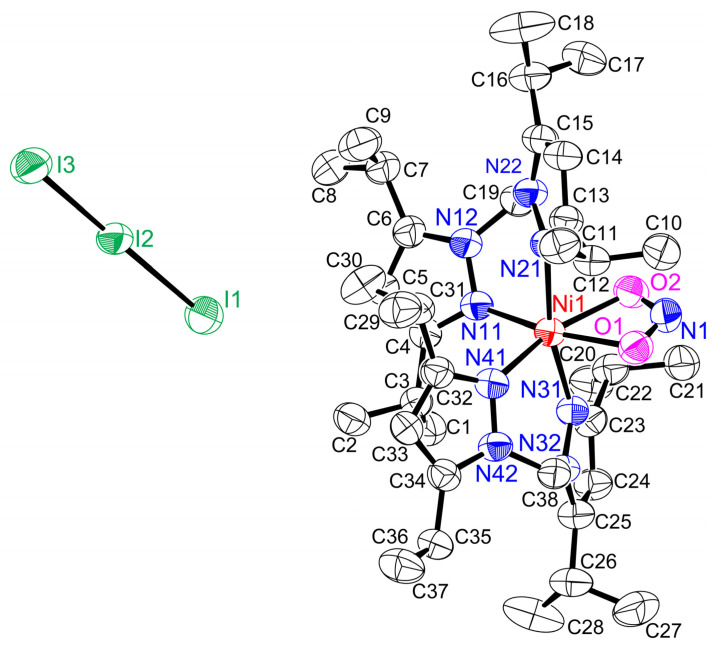
Molecular structure of **[Ni(κ^2^-O_2_N)(L1″)_2_](I_3_)** showing 50% thermal ellipsoids and the atom-labeling scheme. Hydrogen atoms are omitted for clarity. Relevant bond lengths (Å) and angles (°): Ni1–N11, 2.102 (3); Ni1–N21, 2.137 (3); Ni1–N31, 2.121 (3); Ni1–N41, 2.125 (3); Ni1–O1, 2.088 (3); Ni1–O2, 2.120 (3); O1–N1, 1.256 (5); O2–N1, 1.268 (5); I1–I2, 2.9433 (6); I2–I3, 2.8693 (7); N11–Ni1–N21, 90.62 (13); N11–Ni1–N31, 93.44 (13); N11–Ni1–N41, 93.83 (13); N21–Ni1–N31, 166.36 (13); N21–Ni1–N41, 99.78 (13); N31–Ni1–N41, 92.94 (13); O1–Ni1–O2, 59.42 (12); O1–Ni1–N11, 165.89 (13); O1–Ni1–N21, 88.43 (13); O–Ni1–N31, 84.46 (12); O1–Ni1–N41, 100.21 (13); O2–Ni1–N11, 106.55 (13); O2–Ni1–N21, 80.40 (13); O2–Ni1–N31, 85.96 (12); O2–Ni1–N41, 159.62 (13); Ni1–O1–N1, 95.5 (2); Ni1–O2–N1, 93.6 (3); O1–N1–O2, 111.5 (3); I1–I2–I3, 176.853 (18).

As mentioned above, the coordinately saturated Ni–NO complexes [Ni(NO)(L3)] and [Ni(NO)(L0f)] do not react with dioxygen. The coordinately unsaturated Ni–NO complex **[Ni(NO)(I)(L1″)]**, on the other hand, reacts with dioxygen at room temperature (Figure 5). During the dioxygen reaction, the solution color changed from green to brown. Time-dependent visible spectral changes for the reaction between **[Ni(NO)(I)(L1″)]** and dioxygen are shown in Figure 6. It is possible to observe that **[Ni(NO)(I)(L1″)]** slowly reacts with dioxygen. We isolated the product and grew crystals. Crystallographic data of the product show that an unexpected six-coordinate structure was obtained, with the oxidation of ^3^NO^−^ and I^−^ to NO_2_^−^ and I_3_^−^, as shown in Figure 7. During the oxidation reaction, **[Ni(NO)(I)(L1″)]** decomposed, and L1″ itself was reconstituted. The product complex has a six-coordinate structure with two L1″ ligands bound to the nickel ion and one coordinated NO_2_^−^, which originates from the oxidation of ^3^NO^−^. The complex is paramagnetic, as evident from ^1^H-NMR spectroscopy, and therefore contains a hs-Ni(II) center.

### 2.5. Structure of Ni-NO_2_ Complex

The structure of **[Ni(κ^2^-O_2_N)(L1″)_2_](I_3_)** is shown in Figure 7 and the relevant bond lengths (Å) and angles (°) are also listed in the figure caption. The bond lengths in I_3_^−^ are I1–I2 = 2.9433 (6) Å and I2–I3 = 2.8693 (7) Å, and the bond angle of I1–I2–I3 is 176.853 (18)°, clearly indicating that I_3_^−^ is asymmetric and not in the fully linear form. This structural behavior of the I_3_^−^ ion is the same as observed in the X-ray structure of CsI_3_, 2.83 Å, 3.03 Å, and 176.3° [38,39]. The coordination geometry in **[Ni(κ^2^-O_2_N)(L1″)_2_](I_3_)** is six-coordinate with four nitrogen atoms from two L1″ coligands and oxygen atoms from bidentate NO_2_^−^ bound to the Ni center. The Ni1–N11, Ni1–N21, Ni1–N31, and Ni1–N41 bond lengths (2.102 (3), 2.137 (3), 2.121 (3), and 2.125 (3) Å) are almost equivalent. The nitrite NO_2_^−^ ligand is in an asymmetric, bidentate coordination mode (Ni1–O1, 2.022 (4) Å and Ni1–O2, 2.120 (3) Å; ∆Ni-O, 0.032 Å) and the O1–Ni1–O2 angle is 111.5 (3)°. Considering the overall charge, the oxidation number of nickel is +II. From this observation, during the oxidation reaction, the oxidation number of the nickel ion remains constant.

### 2.6. Characterizations of Ni-NO_2_ Complex

**Figure 8:**
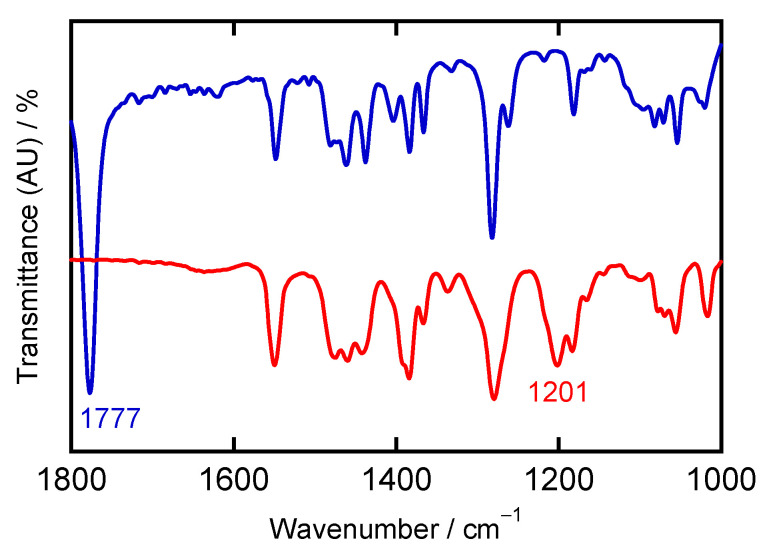
IR spectra of **[Ni(NO)(I)(L1″)]** (blue) and **[Ni(κ^2^-O_2_N)(L1″)_2_](I_3_)** (red), expanding the 1800–1000 cm^−1^ region. The full range (4000–400 cm^−1^) of both IR are provided in Figure S1 (**[Ni(NO)(I)(L1″)]**) and Figure S8 (**[Ni(κ^2^-O_2_N)(L1″)_2_](I_3_)**).

IR and far-IR spectra of **[Ni(κ^2^-O_2_N)(L1″)_2_](I_3_)** were measured using KBr and CsI pellets, respectively (Figures S8–S10 and Figure 8). During the oxidation reaction, the characteristic ν(N–O) signal of the NO complex at 1777 cm^−1^ disappeared. A new signal appeared at 1201 cm^−1^, which can be attributed to one of the ν(N–O) stretches of the coordinated NO_2_^−^ group (Figure 8) and which is consistent with the X-ray structure of this compound, as shown in Figure 7 and Figure S8. In our previous results, the symmetric ν_s_(N–O) stretch of the NO_2_^−^ group in [Cu(κ^2^-ONO)(L3)] was observed at 1264 cm^−1^ [24]. The assignment of the 1201 cm^−1^ band to an N-O stretch of nitrite was also confirmed by the isotopic labeling experiment, using ^18^O_2_ in the oxidation reaction (Figure S10). In this case, the 1201 cm^−1^ band has shifted to lower energy, into the region of the 1182 cm^−1^ band, and is therefore located around 1180 cm^−1^ (the exact peak position cannot be determined). The ν(N–^16^O/^18^O) shift is in agreement with half of the value (19 cm^−1^ = 1201–1182) of the shift (32 cm^−1^) calculated using the reduced mass, assuming two ^18^O are incorporated in the NO_2_^−^ group. This is consistent with the solution state reactivity, which shows one oxygen atom from O_2_ being incorporated into the NO_2_^−^ group [20,21,36,40]. The exact assignment of the 1201 cm^−1^ feature is further discussed in the computational section below. In the far-IR spectrum of **[Ni(κ^2^-O_2_N)(L1″)_2_](I_3_)**, a strong peak is observed at 137 cm^−1^, which can be assigned to the ν_3_ band of the triiodide anion [ν_as_(I–I)] (Figure S9) [39,41,42]. This assignment is confirmed by the far-IR band of CsI_3_, observed at 149 cm^−1^ [39].

**Figure 9:**
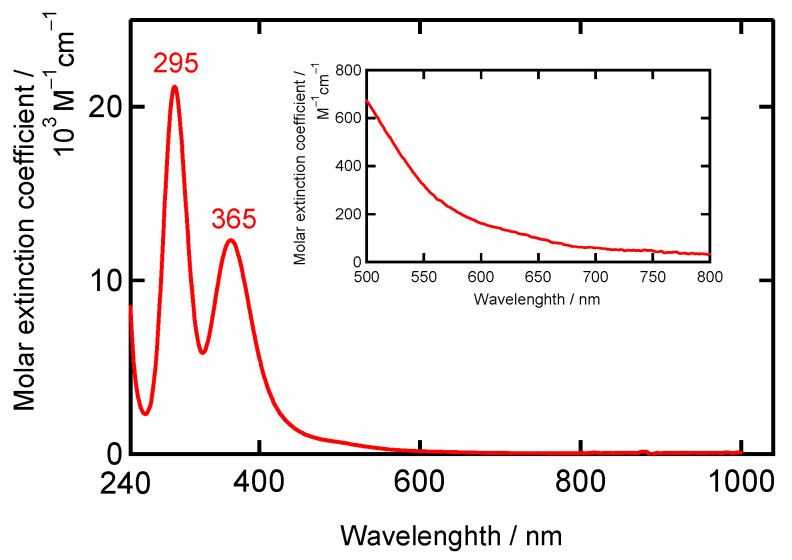
UV–Vis spectrum of **[Ni(κ^2^-O_2_N)(L1″)_2_](I_3_)** in CH_2_Cl_2_. Inset: expanding the 350–800 nm region.

UV–Vis spectra in the solution state (dichloromethane solution) and the solid state (Nujol) are shown in Figure 9 and Figure S11. The similarities of the UV–Vis spectra in both states indicate that the complex retains its structure in the solution state, similar to the crystal (solid state) structure. Characteristic strong bands in the UV region are observed at 295 nm (21,100 M^−1^cm^−1^) and 365 nm (12,300 M^−1^cm^−1^) (Figure 9). Both bands are assigned to σ → σ* and π → σ* transitions of the asymmetrical I_3_^−^ anion [41,42,43,44]. From these strong absorption bands, this obtained oxygenated complex **[Ni(κ^2^-O_2_N)(L1″)_2_](I_3_)** shows a brown color. It is very difficult to assign the *d*–*d* bands in the distorted octahedral geometry of **[Ni(κ^2^-O_2_N)(L1″)_2_](I_3_)**, as these features have low intensity and are partially masked by the intense I_3_^−^ transitions. 

### 2.7. Theoretical Considerations

#### 2.7.1. Density Functional Theory (DFT) Calculations for **[Ni(NO)(I)(L1″)]**

DFT calculations were performed for the **[Ni(NO)(I)(L1″)]** complex using def2-TZVP as the basis set and different functionals. The results are shown in Table 2. For the functionals used in this work, we see that all underestimate the Ni–NO distance and the Ni–N–O angle, while the N–O distance and the ν(N–O) and ν(Ni–NO) frequencies are overestimated. Overall, the geometry and frequencies are more accurately predicted by the BP86 method, whereas the functionals with exact exchange overestimate the vibrational frequencies, especially ν(N–O), quite substantially. Similar observations have previously been made, not only for hs-{NiNO}^10^, but for other {MNO}^x^ systems as well [45]. Compared to [Ni(NO)(L3)] [22], both the experimental and the computational results show that in both hs-{NiNO}^10^ complexes, the NiNO units have very similar geometric and electronic properties (see Table 2).

**Figure 10:**
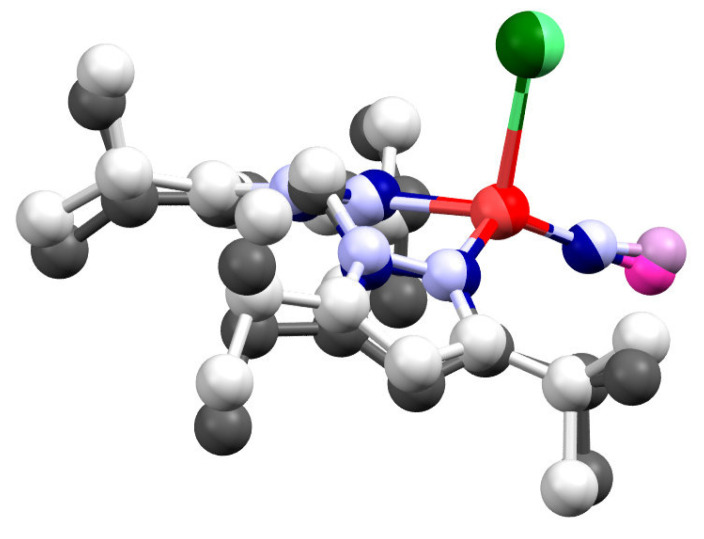
Superposition of the crystal structure (dark colors) and the optimized structure (light colors) of **[Ni(NO)(I)(L1″)]** in the broken symmetry state using the B3LYP functional. Hydrogen atoms are removed for clarity. Color: nickel: red, nitrogen: blue, oxygen: magenta, iodine: green, and carbon: gray/black.

In these calculations, a closed-shell (CS) electron configuration was used. However, experimentally, the related complex [Ni(NO)(L3)] has been shown to have an open-shell ground state, with a hs-Ni(II) center (*S* = 1) bound to a triplet NO^−^ ligand (*S* = 1), and antiferromagnetically coupled spins (total spin *S*_total_ = 0) [22]. Nevertheless, the broken-symmetry (BS) ground state could not be geometry optimized for this complex. For **[Ni(NO)(I)(L1″)]**, we therefore investigated whether the analogous open-shell singlet electronic state could be geometry optimized. Excitingly, using B3LYP we were able to obtain a fully optimized structure of the complex in the BS state. The resulting wavefunction shows spin densities of +1.05 on Ni(II) and −1.19 on the NO^−^ ligand. A superposition of the calculated BS structure and the crystal structure, shown in Figure 10, shows overall a good agreement. 

Comparing the results obtained for the open-shell (BS) and CS systems using the B3LYP functional, we see that for the BS state, the Ni–NO bond distance is longer and ν(Ni–NO) is lower compared to experiment. In contrast, for the CS state, the Ni–NO distance is underestimated and ν(Ni–NO) is overestimated. These results show that the experimental Ni–NO bond strength is in between the predictions for the open-shell and closed-shell system. On the other hand, the Ni–N–O bond angle and the N–O stretching frequency obtained for the BS state are closer to the experimental values. The calculated energy for **[Ni(NO)(I)(L1″)]** in the BS state is about 2 kcal/mol lower compared to the CS system. Given the similarities between the structural and spectroscopic properties of **[Ni(NO)(I)(L1″)]** and [Ni(NO)(L3)] and the detailed investigations on the latter complex [22], these results are consistent with the open-shell, antiferromagnetically coupled ground state for **[Ni(NO)(I)(L1″)]**, as observed for [Ni(NO)(L3)]. Attempts to geometry-optimize the complex in the BS state using BP86 were not successful, as the wavefunction always collapsed into the CS state. 

#### 2.7.2. DFT Calculations for **[Ni(κ^2^-O_2_N)(L1″)_2_]^+^**

In order to assist with the experimental findings, DFT calculations were also performed for the **[Ni(κ^2^-O_2_N)(L1″)_2_]^+^** product complex, which is obtained after the reaction of **[Ni(NO)(I)(L1″)]** with O_2_. In this case, the complex does not involve an antiferromagnetically coupled ground state. For this reason, optimization and frequency calculations were performed using the functional BP86, since it shows the overall best results for the Ni–NO complex. The results from these calculations are shown in Table 3.

Comparison between the experimental and DFT-calculated structures for the product shows an overestimate of the Ni–O distances in the BP86 calculation, while the O–N–O angle of nitrite is reproduced with high accuracy. The calculated symmetric N–O stretching frequency of nitrite is predicted at 1256 cm^−1^, which shift to 1247/1241 cm^−1^ upon ^18^O isotope labeling (with only one O atom of nitrite labeled; see above). On the other hand, the antisymmetric N–O stretch of coordinated nitrite is predicted at 1167 cm^−1^, shifting to 1147 cm^−1^ with ^18^O. The experimental value for the N–O stretch is observed at 1201 cm^−1^, which is right in between those predicted frequencies. However, the experimentally observed isotope shift of 19 cm^−1^ (1201 to 1182 cm^−1^, Figure S10) matches that of the antisymmetric stretch, which also has the larger calculated IR-spectral intensity. We therefore assign the N–O stretch observed experimentally to the antisymmetric mode. 

## 3. Materials and Methods

### 3.1. Material and General Techniques

The preparation and handling of the two nickel(II) complexes were performed under an argon atmosphere using standard Schlenk tube techniques. Diethyl ether, tetrahydrofuran (THF), and *n*-heptane were distilled from sodium benzophenone ketyl under an argon atmosphere. Dichloromethane was purified by distillation from phosphorous pentoxide under an argon atmosphere [46]. Deuterated solvents were obtained from Cambridge Isotope Laboratories, Inc. (Tewksbury, MA, USA). NO gas and ^16^O_2_ gas were purchased from TOKAI (Ibaraki, Japan), and NO gas was purified by passing through a column filled with solid NaOH before the reaction. ^18^O_2_ gas was obtained from Shoko Science and was used without any purification (Yokohama, Japan). Other reagents were commercially available and used without further purification. The bis(3,5-diisopropyl-1-pyrazolyl)methane (L1″) was prepared following a published method [24]. The purity of the ligand was checked by ^1^H-NMR and IR spectroscopy.

### 3.2. Instrumentation

IR spectra (4000–500 cm^−1^) and far-IR spectra (700–120 cm^−1^) were recorded as KBr pellets using a JASCO FT/IR-6300 spectrophotometer under ambient conditions (JASCO, Tokyo, Japan) and as CsI pellets using a JASCO FT/IR 6700 spectrophotometer under vacuum (JASCO, Tokyo, Japan), respectively. Abbreviations used in the description of vibrational data are as follows: vs, very strong; s, strong; m, medium; w, weak. ^1^H-NMR (500 MHz) and ^13^C-NMR (125 MHz) spectra were obtained on a Bruker AVANCE III-500 NMR spectrometer at room temperature (298 K) in CDCl_3_-*d*_1_ or (CD_3_)_2_CO-*d*_6_ (Bruker Japan, Yokohama, Japan). ^1^H and ^13^C chemical shifts were reported as *^TM^* values relative to residual solvent peaks. UV–Vis spectra (solution and solid, 1040–240 nm) were recorded on a JASCO V-570 spectrophotometer (JASCO, Tokyo, Japan). Solid samples (mulls) for UV–Vis spectroscopy were prepared by finely grinding microcrystalline material into powders with a mortar and pestle and then adding mulling agents (Nujol, poly(dimethylsiloxane), viscosity 10,000) (Aldrich, Milwaukee, WI, USA)) before uniformly spreading between quartz plates equipped with an integrating sphere apparatus (JASCO ISN-470 Tokyo, Japan) using fine powder samples. Time-dependent spectral changes in UV–Vis spectroscopy were recorded on a JASCO V-570 spectrophotometer (JASCO, Tokyo, Japan) under dioxygen atmosphere. This solution was kept at room temperature and stirred by small magnetic stirrer bar at 200 revolutions per min by thermal controller (JASCO ETC-505T, Tokyo, Japan). Absorption spectral changes were measured at 60 min intervals for 120 h. The elemental analyses (C, H, and N) were performed by the Chemical Analysis Center of Ibaraki University.

### 3.3. Theoretical Calculations

Computational studies were performed using Gaussian 16 software [47]. The geometries of the complexes studied here were optimized without any restraints. The initial structure for **[Ni(NO)(I)(L1″)]** was obtained from crystallographic data by removing the tetrahydrofuran molecule present in the structure. The initial structure for **[Ni(κ^2^-O_2_N)(L1″)_2_]** was also obtained from the crystal structure, this time by removing the triiodine anion. All atoms in the structure were treated using the triple-ζ polarized basis set of Ahlrichs and coworkers (def2-TZVP) [48,49]. The calculations were performed using the following functionals: (i) Becke’s 1988 exchange functional [50] and the gradient corrections of Perdew, along with his 1981 local correlation functional P86 (BP86) [51]; (ii) Becke’s three-parameter hybrid exchange functional [52], along with the gradient corrections provided by the Lee–Yang–Parr nonlocal correlation functional (B3LYP) [53]. For **[Ni(NO)(I)(L1″)]**, we performed both closed-shell and broken-symmetry calculations.

### 3.4. Preparation of Ligand and Complexes

#### 3.4.1. [Ni(NO)(I)]*_n_*

This complex was prepared using a modified version of the reported method [35]. To remove water, the starting nickel powder of 1.050 g (17.9 mmol) was settled in an oil bath at 120 °C for 5 h. After cooling to room temperature, acetone (20 mL) was added, and the mixture was stirred for 5 min. To this solution, an ether solution (30 mL) of iodine (1.556 g, 6.13 mmol) was added dropwise over 1 h. About 200 cm^3^ of NO gas was passed through the solution, and the mixture was stirred at room temperature for 2 h, which was then stirred for another 0.5 h after one more NO gas addition (200 cm^3^). After the reaction, the solvent was removed under vacuum and the dark green solid was obtained (3.02 g (14.0 mmol, yield 88%)).

IR (KBr)/cm^−1^ (assignment): 1854 vs (Ni–NO), 1635 w, 1261 w, 1084 w, 799 w. UV–Vis (solid, Nujol) *λ*_max_/nm): 437, 802.

#### 3.4.2. **[Ni(NO)(I)(L1″)]**

To a solution of [Ni(NO)(I)]*_n_* (0.0967 g, 0.449 mmol) in tetrahydrofuran (10 mL) was added L1” (0.1346 g, 0.425 mmol) in dichloromethane (10 mL). The resulting solution was stirred at room temperature for 3 h. After that, the obtained green solution was filtered off using Celite to remove unreacted nickel powder. The solvent was removed under vacuum. Recrystallization from dichloromethane/*n*-heptane at −30 °C gave green crystals. Single crystals suitable for X-ray diffraction were obtained by slow recrystallization under the same experimental conditions. Yield: 53% (0.1194 g, 0.224 mmol). 

Anal. Calcd for C_19_H_32_IN_5_NiO·0.5(H_2_O): C 42.89, H 6.06, N 13.16. Found: C 41.94, H 5.87, N 12.77. IR (KBr/cm^−1^): 2965 s, 2933 m, 2869 s, 1776 vs *ν*(N–O), 1547 m, 1461 m, 1437 m, 1402 w, 1382 m, 1366 w, 1281 s, 1181 m, 1053 m, 809 m, 796 m. Far-IR (CsI, cm^−1^): 661 s, 571 m, 543 w, 470 w, 410 w *δ*(Ni–N–O), 381 m, 329 w, 303 w, 252 w, 212 w, 188 w, 163 w. ^1^H NMR (CDCl_3_) *δ*/ppm (assignment): 1.25 (s, broad, 12H, CH(C*H*_3_)_2_), 1.40 (s, broad, 6H, CH(C*H*_3_)_2_), 1.67 (s, broad, 6H, CH(C*H*_3_)_2_), 2.75 (sep, *J* = 7.0 Hz, 2H, C*H*(CH_3_)_2_), 3.83 (sep, *J* = 7.0 Hz, 2H, C*H*(CH_3_)_2_), 4.02 (s, 2H, C*H*_2_), 6.24 (s, 2H, 4–pz). ^1^H NMR (acetone-*d*_6_) *δ*/ppm (assignment): 1.30 (s, broad, 12H, CH(C*H*_3_)_3_), 1.47 (s, broad, 6H, CH(C*H*_3_)_3_), 1.71 (s, broad, 6H, CH(C*H*_3_)_3_), 3.23 (sep, *J* = 7.0 Hz, 2H, C*H*(CH_3_)_3_), 3.85 (sep, *J* = 7.0 Hz, 2H, C*H*(CH_3_)_3_), 3.95 (s, 2H, C*H*_2_), 6.60 (s, 2H, 4–pz). ^13^C NMR (CDCl_3_) *δ*/ppm (assignment): 22.9 (CH(*C*H_3_)_2_), 25.7 (CH(*C*H_3_)_2_), 29.2 (*C*H(CH_3_)_2_), 53.1 (H_2_*C*), 100.6 (pz-4*C*), 152.4 (pz-3*C*), 164.7 (pz-5*C*). UV–Vis (solution, CH_2_Cl_2_) *λ*_max_/nm (*ε*/M^−1^cm^−1^): 290 (4060), 375 (340), 492 (130), 717 (400). UV–Vis (solid, Nujol) *λ*_max_/nm: 297, 383, 500, 646, 730.

#### 3.4.3. **[Ni(κ^2^-O_2_N)(L1″)_2_](I_3_)**

In a 50 mL Schlenk tube, **[Ni(NO)(I)(L1″)]** (0.0476 g, 0.089mmol) was dissolved in dichloromethane (9 mL) at room temperature in an argon atmosphere. The argon was then replaced with O_2_ gas and the solution was stirred at room temperature for 3 days. During this time, the color of the solution changed from deep green to brown. Recrystallization from dichloromethane/*n*-heptane at −30 °C gave brown crystals. Single crystals suitable for X-ray diffraction were obtained by slow recrystallization under the same experimental conditions. Yield: 45% (0.0226 g, 0.0202 mmol). 

Anal. Calcd for C_38_H_64_I_3_N_9_NiO_2_: C 40.81, H 5.77, N 11.27. Found: C 40.67, H 5.77, N 11.27. IR (KBr)/cm^−1^(assignment): 3123 w, 2967 vs, 2930 s, 2870 m, 1549 s, 1475 s, 1459 s, 1442 s, 1383 s, 1366 m, 1336 w, 1279 vs, 1201 s(*ν*(NO_2_)), 1183 s, 1055 m, 1016 w, 812 w, 661 w. Far-IR (CsI) cm^−1^: 672s, 662 s, 584 w, 539 m, 508 w, 475 w, 413 w, 391 w, 320 w, 311 w, 228 w, 182 w, 138 vs. UV–Vis (solution, CH_2_Cl_2_) *λ*_max_/nm (*ε*/M^−1^cm^−1^): 295 (21,100), 365 (12,300). UV–Vis (solid, Nujol) *λ*_max_/nm: 301, 376.

For the ^18^O_2_ reaction, **[Ni(NO)(I)(L1″)]** (0.0107 g, 0.0201 mmol) was dissolved in dichloromethane (5 mL) at room temperature in an argon atmosphere. The argon was then replaced with ^18^O_2_ gas and the solution was stirred at room temperature for 3 days. After the reaction, the recrystallization was carried out using the above procedure.

IR (KBr)/cm^−1^(assignment): 3125 w, 2968 vs, 2931 s, 2870 m, 1550 m, 1473 m, 1459 m, 1441 m, 1385 s, 1366 m, 1335 w, 1280 vs (*ν*(N^18^O^16^O)), 1182 m, 1095 w, 1079 m, 1055 m, 1018 m, 810 m, 661 w.

### 3.5. X-ray Crystal Structure Determination

The diffraction data of **[Ni(NO)(I)(L1″)]** and **[Ni(κ^2^-O_2_N)(L1″)_2_](I_3_)** were obtained on a Rigaku XtaLAB P200 diffractometer using multilayer mirror monochromated MoKα (λ = 0.71073 Å) radiation at −95 ± 2 °C. A crystal of suitable size and quality was coated with Paratone-N oil (Hampton Research, Aliso Viejo, CA, USA) and mounted on a Dual-Thickness MicroLoop LD (200 µM) (MiTeGen, New York, NY, USA). The unit cell parameters were determined using *CrystalClear* from 18 images [54]. The crystal to detector distance was ca. 45 mm. Data were collected at 0.5° intervals in φ and ω to a maximum 2θ value of 55.0°. The highly redundant datasets were reduced using *CrysAlisPro* [55]. An empirical absorption correction was applied for each complex. Structures were solved by direct methods (*SIR2008* [56]). The position of the silver ions and their first coordination sphere were located using a direct method (*Emap*). Other nonhydrogen atoms were found in alternating difference Fourier syntheses, and least squares refinement cycles. During the final refinement cycles, the temperature factors were refined anisotropically. Refinement was carried out by a full matrix least squares method on *F*^2^. All calculations were performed with the *CrystalStructure* [57] crystallographic software package except for refinement, which was performed using *SHELXL 2013* [58]. Hydrogen atoms were placed in calculated positions. Crystallographic data and structure refinement parameters, including the final discrepancies (*R* and *R_w_*), are listed in Table 4.

## 4. Conclusions

Previously, we reported a Ni–NO complex with a hindered N3 type coligand, [Ni(NO)(L3)], where L3^−^ denotes hydrotris(3-tertiary butyl-5-isopropyl-1-pyrazolyl)borate [22]. This complex is very stable toward dioxygen. In this research, we explored the dioxygen reactivity of the Ni–NO complex **[Ni(NO)(I)(L1″)]** with the less hindered N2 type bis(pyrazolyl)methane coligand L1″, to create a coordinatively unsaturated ligand environment, where L1″ denotes bis(3,5-diisopropyl-1-pyrazolyl)methane. The coordination geometry in **[Ni(NO)(I)(L1″)]** is four-coordinate with three nitrogen atoms from L1″ and NO and a bound iodide anion. We could conclude that the coordination geometry about the Ni center is distorted tetrahedral, based on distances between the basal planes and the metal ion. The ν(N–O) value is 1777 cm^−1^ in **[Ni(NO)(I)(L1″)]**, which is clearly shifted from ν(N–O) in the starting material [Ni(NO)(I)]*_n_*, observed at 1854 cm^−1^. Moreover, the Ni–NO stretching vibration, ν(Ni–NO), is observed at 572 cm^−1^. This assignment is based on the consideration of the vibrational frequency of ν(Ni–NO) in related complexes, especially [Ni(NO)(L3)]. Considering the electron-donating properties of hydrotris(pyrazolyl)borate type ligands, the overall electronic structure of the related complexes [Ni(NO)(L3)] and **[Ni(NO)(I)(L1″)]** is almost the same, in spite of the different coordinating groups (pyrazolyl nitrogen versus iodide anion) and different ligand charges (neutral versus minus one). From the UV–Vis assignments, the Ni oxidation state is +II, and the Ni(II) center is in the high-spin state. This implies that the NO ligand is ^3^NO^−^ (nitroxyl) in the complex, since the complex charge is zero, which is analogous to the electronic structure reported for [Ni(NO)(L3)]. In comparison, **[Ni(NO)(I)(L1″)]** has a green color, not blue like [Ni(NO)(L3)]. This difference relates to the different ligand donor set (N3I1 versus N4), causing a shift in the low-energy d–d transitions in **[Ni(NO)(I)(L1″)]**.

After dioxygen reaction of **[Ni(NO)(I)(L1″)]**, we obtained an unexpected six-coordinate compound, **[Ni(κ^2^-O_2_N)(L1″)_2_](I_3_)**, as the product where both the NO and the I^−^ ion were oxidized to yield NO_2_^−^ and I_3_^−^. A rearrangement of the L1″ coligand was observed as well, with the product complex having two L1″ coligands bound. Considering the overall charge, the oxidation number of nickel is +II. Therefore, during this oxidation reaction, the oxidation number of the nickel ion remains constant. Moreover, a new stretching vibration appeared at 1201 cm^−1^ upon exposure of **[Ni(NO)(I)(L1″)]** to O_2_, which can be attributed to one of the ν(N–O) modes of the bound NO_2_^−^ group. For the anion I_3_^−^, the ν_as_(I–I) band was observed at 137 cm^−1^, and characteristic strong bands in the UV region of the absorption spectrum were observed at 295 nm (21,100 M^−1^cm^−1^) and 365 nm (12,300 M^−1^cm^−1^), which are assigned as σ → σ* and π → σ* transitions of I_3_^−^, respectively.

Theoretical calculations for **[Ni(NO)(I)(L1″)]** using both closed-shell and broken-symmetry electronic states were conducted using different DFT methods. Notably, using B3LYP, we were able to optimize the structure of the complex for both of these electronic states, allowing us to make direct comparisons. The results suggest that the complex is best described as a hs-Ni(II) center (*S* = 1) antiferromagnetically coupled to the ^3^NO^−^ ligand (*S* = 1; giving a total spin *S*_total_ = 0), with the broken-symmetry state being about 2 kcal/mol lower in energy than the closed-shell state. The electronic structure of **[Ni(NO)(I)(L1″)]** is therefore analogous to that of [Ni(NO)(L3)] reported previously, in agreement with their very similar structural and spectroscopic properties. DFT calculations for the six-coordinate product complex **[Ni(κ^2^-O_2_N)(L1″)_2_]^+^** were also performed. Our results suggest that the N–O stretching mode of nitrite, observed experimentally at 1201 cm^−1^, corresponds to the antisymmetric stretch, predicted at 1167 cm^−1^ with a calculated ^18^O isotope shift of about 20 cm^−1^, in agreement with experiment.

## Figures and Tables

**Table 1:** Representative data of Ni–NO complexes with tripodal coligands related to this work.

Complexes ^a^	Ligand Type	*d* (Ni–N)/Å ^b^	*d* (N–O)/ Å ^b^	∠ (Ni–N–O)/ ° ^b^	ν(N–O)/ cm^−1 b^	{MNO}*^n^*	Ref.
**[Ni(NO)(I)(L1″)]**	(NO)N2I	1.6467 (16)	1.136 (3)	176.68 (19)	1777 (KBr)	{NiNO}^10^	tw ^c^
[Ni(NO)(L3)]	(NO)N3	1.651 (6)	1.160 (10)	179.1 (7)	1780 (KBr)	{NiNO}^10^	[22]
[Ni(NO)(L0f)]	(NO)N3	1.6361 (16)	1.160 (2)	172.42 (17)	1823 (KBr)	{NiNO}^10^	[27]
[Ni(NO)(L0)]	(NO)N3	1.619 (6), 1.617 (6)	1.170 (7), 1.158 (7)	178.5 (6), 175.3 (7)	1786 (KBr)	na ^d^	[28]
						{NiNO}^10^	[29]
[Ni(NO)(iPr_3_tacn)](PF_6_)	(NO)N3	1.677 (4) 1.646 (3)	1.123 (5) 1.162 (4)	180.0 170.9 (2)	1770 (ATR)	{NiNO}^10^	[30]
[Ni(NO)(Tm*^p^*^-tol^)]	(NO)S3	1.665 (3)	1.131 (4)	173.9 (4)	1752 (KBr)	{NiNO}^10^	[31]
[Ni(NO)(Tse^Mes^)]	(NO)Se3	1.633 (4)	1.156 (5)	180.0	1763, 1752 (KBr)	na ^d^	[28]
[Ni(NO){HB(*^t^*BuIm)_3_}]	(NO)C3	1.620 (5)	1.184 (7)	178.5 (4)	1703 (toluene)	{NiNO}^10^	[32]
[Ni(NO){PhB(CyCH_2_Im)_3_}]	(NO)C3	1.633 (9)–1.668 (1)	1.174 (1)–1.197 (1)	172.7 (1)–177.8 (1)	1693 (THF)	{NiNO}^10^	[33]
[Ni(NO){HB(MeBz)_3_}]	(NO)C3	1.643 (2)–1.646 (2)	1.183 (3)–1.191 (3)	169.3 (2)–174.8 (2)	1714 (THF)	{NiNO}^10^	[33]
[Ni(NO){HB(*p*-^t^BuPhTz)_3_}]	(NO)C3	1.640 (2)	1.163 (3)	176.3 (3)	1746 (THF)	{NiNO}^10^	[33]

^a^ Full names of the coligands are noted in the main text. ^b^ Highest and lowest values are marked in red. ^c^ tw denotes “this work”. ^d^ na denotes “not available”.

**Table 2:** Comparison of geometries, vibrational frequencies, and electronic aspects of **[Ni(NO)(I)(L1″)]** between experiment and DFT calculations using different functionals.

Method ^a^	*d* (Ni–N) /Å	*d* (N–O) /Å	∠ (Ni–N–O) /°	ν(N–O) /cm^−1^	ν(Ni–NO) /cm^−1^	δ(Ni–N–O) /cm^−1^	SD ^b^ (Ni)	SD ^b^ (NO)
**Exp.**	1.647	1.135	176.7	1777	572	410		
**BP86 (CS)**	1.646	1.172	165.1	1799	618	410	0	0
**B3LYP (CS)**	1.630	1.156	163.8	1883	651	412	0	0
**B3LYP (BS)**	1.720	1.169	167.1	1820	439	356	1.05	−1.19

^a^ CS = closed shell, BS = broken symmetry. ^b^ SD = spin density.

**Table 3:** Comparison of geometries and vibrational frequencies for **[Ni(κ^2^-O_2_N)(L1″)_2_]^+^** (*S*_total_ = 1) between experiment and DFT calculations using the BP86 functional.

Method	*d* (Ni–O1) /Å	*d* (Ni–O2) /Å	*d* (N1–O1) /Å	*d* (N1–O2) /Å	∠ (O1–N1–O2) /°	ν_as_(O–N–O) /cm^−1^
**Exp.**	2.088	2.120	1.256	1.267	111.5	1201
**BP86**	2.178	2.107	1.268	1.274	111.8	1166

**Table 4:** Crystal data and structure refinement of **[Ni(NO)(I)(L1″)](thf)** and **[Ni(κ_2_-NO_2_)(L1″)_2_](I_3_)**.

Complex	[Ni(NO)(I)(L1″)](thf)	[Ni(κ_2_-NO_2_)(L1″)_2_](I_3_)
CCDC number	2285305	2285306
Empirical formula	C_23_H_40_IN_5_NiO_2_	C_38_H_64_I_3_N_9_NiO_2_
Formula weight	604.21	1118.40
Crystal system	Triclinic	Monoclinic
Space group	*P*$\bar{1}$ (#2)	*P*2_1_/*n* (#14)
*a*/Å	9.02661 (11)	9.3444 (2)
*b*/Å	9.61413 (11)	17.2498 (3)
*c*/Å	16.4456 (2)	30.0610 (5)
*α*/°	77.6870 (10)	90
*β*/°	89.2370 (10)	86.7699 (17)
*γ*/°	87.2540 (10)	90
*V*/Å^3^	1392.76 (3)	4837.81 (16)
*Z*	2	4
*D*_calc_/g cm^−3^	1.441	1.535
*μ*(MoKα)/cm^−1^	18.318	23.555
2*θ*_range,_ °	6–55	6–55
Reflections collected	45,524	115,535
Unique reflections	6385	10,976
*R*_int_	0.0335	0.0280
Number of variables	289	478
Refls./Para. ratio	22.09	22.96
Residuals: *R*1 (*I* > 2 σ (*I*)) ^a^	0.0233	0.0747
Residuals: *R* (All refl.)	0.0247	0.0843
Residuals: *wR*2 (All refl.) ^a^	0.0653	0.1898
Goodness of fit ind.	1.064	1.103
Max/min peak, /e Å^−3^	0.76/−0.30	2.46/−1.05

^a^ *R*1 = Σ ||*Fo*|−|*Fc*||/Σ |*Fo*|; *wR*2 = [{Σ[*w*(|*Fo*|^2^−|*Fc*|^2^)^2^]/Σ*w*(*Fo*^2^)}^2^]^1/2^.

## Data Availability

The crystallographic data are available from the Cambridge Crystallographic Data Centre (CCDC).

## Supplementary Materials

The following are available online at https://www.mdpi.com/article/10.3390/molecules28176206/s1, CIF files. Figure S1: IR spectrum of **[Ni(NO)(I)(L1″)]** (KBr); Figure S2: Far-IR spectrum of **[Ni(NO)(I)(L1″)]** (CsI); Figure S3: IR and UV–Vis spectra of the starting material [Ni(NO)(I)]*_n_*; Figure S4: UV–Vis spectrum of **[Ni(NO)(I)(L1″)]** in solid state (Nujol); Figure S5: ^1^H-NMR spectrum of **[Ni(NO)(I)(L1″)]** in CDCl_3_-*d*_1_; Figure S6: ^1^H-NMR spectrum of **[Ni(NO)(I)(L1″)]** in (CD_3_)_2_CO-*d*_6_; Figure S7: ^13^C-NMR spectrum of **[Ni(NO)(I)(L1″)]** in CDCl_3_-*d*_1_; Figure S8: IR spectrum of **[Ni(κ^2^-NO_2_)(L1″)_2_](I_3_)** (KBr); Figure S9: Far-IR spectrum of **[Ni(κ^2^-NO_2_)(L1″)_2_](I_3_)** (KBr); Figure S10: IR spectral comparisons of the reaction of **[Ni(NO)(I)(L1″)]** with ^16^O_2_ and ^18^O_2_; Figure S11: UV–Vis spectrum of **[Ni(κ^2^-O_2_N)(L1″)_2_](I_3_)** in the solid state (Nujol); Table S1: XYZ coordinates for the optimized structure of **[Ni(NO)(I)(L1″)]** using the BP86 functional for the closed-shell system; Table S2: XYZ coordinates for the optimized structure of **[Ni(NO)(I)(L1″)]** using the B3LYP functional for the closed-shell system; Table S3: XYZ coordinates for the optimized structure of **[Ni(NO)(I)(L1″)]** using the B3LYP functional for the broken-symmetry system; Table S4: XYZ coordinates for the optimized structure of **[Ni(κ^2^-O_2_N)(L1″)_2_]** using the BP86 functional.

## Sample Availability

Samples of the nickel(II) compounds described in this study are available from the authors.

## References

[1] Feldman P.L., Griffith O.W., Stuehr D.J. (1993). The surprising life of nitric oxide. Chem. Eng. News.

[2] Beckman J.S., Koppenol W.H. (1996). Nitric oxide, superoxide, and peroxynitrite: The good, the bad, and the ugly. Am. J. Physiol..

[3] Ignarro L. (2000). Nitric Oxide: Biology and Pathobiology.

[4] Li L., Li L. (2016). Recent advances in multinuclear metal nitrosyl complexes. Coord. Chem. Rev..

[5] Maia L.B., Moura J.J.G. (2014). How biology handles nitrite. Chem. Rev..

[6] Tsai M.-L., Tsou C.-C., Liaw W.-F. (2015). Dinitrosyl iron complexes (DNICs): From biomimetic synthesis and spectroscopic characterization toward unveiling the biological and catalytic roles of DNICs. Acc. Chem Res..

[7] Lehnert N., Fujisawa K., Camarena S., Dong H.T., White C.J. (2019). Activation of non-heme iron-nitrosyl complexes: Turning up the heat. ACS Catal..

[8] Ferousi C., Majer S.H., DiMucci I.M., Lancaster K.M. (2020). Biological and bioinspired inorganic N–N bond-forming reactions. Chem. Rev..

[9] Pauleta S.R., Carepo M.S.P., Moura I. (2019). Source and reduction of nitrous oxide. Coord. Chem. Rev..

[10] Lehnert N., Kim K., Dong H.T., Harland J.B., Hunt A.P., Manickas E.C., Oakley K.M., Pham J., Reed G.C., Alfaro V.C. (2021). The biologically relevant coordination chemistry of iron and nitric oxide: Electronic structure and reactivity. Chem. Rev..

[11] Jørgensen C.K. (1966). Differences between the four halide ligands, and discussion remarks on trigonal-bipyramidal complexes, on oxidation states, and on diagonal elements of one-electron energy. Coord. Chem. Rev..

[12] Kaim W., Schwederski B. (2010). Non-innocent ligands in bioinorganic chemistry—An overview. Coord. Chem. Rev..

[13] Mingos D.M.P. (2014). A review of complexes of ambivalent and ambiphilic Lewis acid/bases with symmetry signatures and an alternative notation for these noninnocent ligands. J. Organomet. Chem..

[14] Enemark J.H., Feltham R.D. (1974). Principles of structure, bonding, and reactivity for metal nitrosyl complexes. Coord. Chem. Rev..

[15] Matsunaga Y., Fujisawa K., Ibi N., Miyashita Y., Okamoto K. (2005). Structural and spectroscopic characterization of first-row transition metal(II) substituted blue copper model complexes with hydrotris(pyrazolyl)borate. Inorg. Chem..

[16] Gorelsky S.I., Basumallick L., Vura-Weis J., Sarangi R., Hodgson K.O., Hedman B., Fujisawa K., Solomon E.I. (2005). Spectroscopic and DFT investigation of [M{HB(3,5-*^i^*Pr_2_pz)_3_}(SC_6_F_5_)] (M = Mn, Fe, Co, Ni, Cu, and Zn) model complexes: Periodic trends in metal–thiolate bonding. Inorg. Chem..

[17] Kitajima N., Fujisawa K., Fujimoto C., Moro-oka Y., Hashimoto S., Kitagawa T., Toriumi T., Tatsumi K., Nakamura A. (1992). A new model for dioxygen binding in hemocyanin. Synthesis, characterization, and molecular structure of the *μ*-*η*^2^:*η*^2^ peroxo dinuclear copper(II) complexes, [Cu(HB(3,5-R_2_pz)_3_)]_2_(O_2_) (R = *i*-Pr and Ph). J. Am. Chem. Soc..

[18] Irving H., Williams R.J.P. (1948). Order of stability of metal complexes. Nature.

[19] Imai S., Fujisawa K., Kobayashi T., Shirasawa N., Fujii H., Yoshimura T., Kitajima N., Moro-oka Y. (1998). ^63^Cu NMR study of copper(I) carbonyl complexes with various hydrotris(pyrazolyl)borates: Correlation between ^63^Cu chemical shifts and CO stretching vibrations. Inorg. Chem..

[20] Fujisawa K., Soma S., Kurihara H., Ohta A., Dong H.T., Minakawa Y., Zhao J., Alp E.E., Hu M.Y., Lehnert N. (2019). Stable ferrous mononitroxyl {FeNO}^8^ complex with a hindered hydrotris(pyrazolyl)borate coligand: Structure, spectroscopic characterization, and reactivity toward NO and O_2_. Inorg. Chem..

[21] Fujisawa K., Soma S., Kurihara K., Dong H.T., Bilodeau M., Lehnert N. (2017). A cobalt–nitrosyl complex with a hindered hydrotris(pyrazolyl)borate coligand: Detailed electronic structure, and reactivity towards dioxygen. Dalton Trans..

[22] Soma S., Stappen C.V., Kiss M., Szilagyi R.K., Lehnert N., Fujisawa K. (2016). Distorted tetrahedral nickel-nitrosyl complexes: Spectroscopic characterization and electronic structure. J. Biol. Inorg. Chem..

[23] Fujisawa K., Tateda A., Miyashita Y., Okamoto K., Paulat F., Praneeth V.N.N., Merkle A., Lehnert N. (2008). Structural and spectroscopic characterization of mononuclear copper(I) nitrosyl complexes: End-on versus side-on coordination of NO to copper(I). J. Am. Chem. Soc..

[24] Lehnert N., Cornelissen U., Neese F., Ono T., Noguchi Y., Okamoto K., Fujisawa K. (2007). Synthesis and spectroscopic characterization of copper(II)-nitrito complexes with hydrotris(pyrazolyl)borate and related coligands. Inorg. Chem..

[25] Fujisawa K., Tada N., Ishikawa Y., Higashimura H., Miyashita Y., Okamoto K. (2004). The most hindered hydrotris(pyrazolyl)borate ligand, X-ray structure of chlorocopper(II) complex: [Cu(Cl){HB(3-Ad-5-Pr*^i^*pz)_3_}] as compared with [Cu(Cl){HB(3-Bu*^t^*-5-Pr*^i^*pz)_3_}]. Inorg. Chem. Commun..

[26] Fujisawa K., Noguchi Y., Miyashita Y., Okamoto O., Lehnert N. (2007). Mononuclear and binuclear copper(I) complexes ligated by bis(3,5-diisopropyl-1-pyrazolyl)methane: Insight into the fundamental coordination chemistry of three-coordinate copper(I) complexes with a neutral coligand. Inorg. Chem..

[27] Fujisawa K., Kataoka T., Terashima K., Tiekink E.R.T. (2022). The crystal structure of nitroxyl-*κN*-{hydridotris(3-trifluoromethyl-5-methylpyrazolyl-1-yl-*κN*^3^)borato}nickel(II), C_15_H_13_BF_9_N_7_NiO. Z. Kristallogr.-N. Cryst. Struct..

[28] Landry V.K., Pang K., Quan S.M., Parkin G. (2007). Tetrahedral nickel nitrosyl complexes with tripodal [N_3_] and [Se_3_] donor ancillary ligands: Structural and computational evidence that a linear nitrosyl is a trivalent ligand. Dalton Trans..

[29] Tomson N.C., Crimmin M.R., Petrenko T., Rosebrugh L.E., Sproules S., Boyd W.R., Bergman R.G., DeBeer S., Toste F.D., Wieghardt K. (2011). A Step beyond the Feltham–Enemark notation: Spectroscopic and correlated ab initio computational support for an antiferromagnetically coupled M(II)–(NO)^–^ description of Tp*M(NO) (M = Co, Ni). J. Am. Chem. Soc..

[30] Griego L., Woods T.J., Mirica L.M. (2022). A five-coordinate Ni(I) complex supported by 1,4,7-triisopropyl-1,4,7-triazacyclononane. Chem. Commun..

[31] Maffett L.S., Gunter K.L., Kreisel K.A., Yap G.P.A., Rabinovich D. (2007). Nickel nitrosyl complexes in a sulfur-rich environment: The first poly(mercaptoimidazolyl)borate derivatives. Polyhedron.

[32] Nieto I., Bontchev R.P., Ozarowski A., Smirnov D., Krzystek J., Telser J., Smith J.M. (2009). Synthesis and spectroscopic investigations of four-coordinate nickel complexes supported by a strongly donating scorpionate ligand. Inorg. Chim. Acta.

[33] Muñoz S.B., Foster W.K., Lin H.-J., Margarit C.G., Dickie D.A., Smith J.M. (2012). Tris(carbene)borate ligands featuring imidazole-2-ylidene, benzimidazol-2-ylidene and 1,3,4-triazol-2-ylidene donors. evaluation of donor properties in four-coordinate {NiNO}^10^ complexes. Inorg. Chem..

[34] Enemark J.H. (1971). Four-coordinate metal nitrosyls. I. the structure of azidonitrosylbis(triphenylphosphine)nickel, Ni(N_3_)(NO)(P(C_6_H_5_)_3_). Inorg. Chem..

[35] Haymore B., Feltham R.D. (1973). Nirosyliron, -cobalt, and -nickel iodides. Inorg. Synth..

[36] Kurihara H., Ohta A., Fujisawa K. (2019). Structures and properties of dinitrosyl iron and cobalt complexes ligated by bis(3,5-diisopropyl-1-pyrazolyl)methane. Inorganics.

[37] Yang L., Powell D.R., Houser R.P. (2007). Structural variation in copper(I) complexes with pyridylmethylamide ligands: Structural analysis with a new four-coordinate geometry index, *τ*_4_. Dalton Trans..

[38] Tasman H.A., Boswijk K.H. (1955). Re-investigation of the crystal structure of CsI_3_. Acta Cryst..

[39] Maki A.G., Forneris R. (1967). Infrared and spectra of some trihalide ions: ICl_2_^−^,IBr_2_^−^, I_3_^−^, I_2_Br^−^, and BrICl^−^. Spectrochim. Acta Part A.

[40] Thyagarajan S., Incarvito C.D., Rheingold A.L., Theopold K.H. (2003). In pursuit of a stable peroxynitrite complex–NO_x_ (x = 1−3) derivatives of Tp^t-Bu,Me^Co. Inorg. Chim. Acta.

[41] Kiefer W., Bernstein H.J. (1972). The UV-laser excited resonance Raman spectrum of the I_3_^–^ ion. Chem. Phys. Lett..

[42] Andrews L., Prochaska E.S., Loewenschuss A. (1980). Resonance Raman and ultraviolet absorption spectra of the triiodide ion produced by alkali iodide-iodine argon matrix reactions. Inorg. Chem..

[43] Kaya K., Mikami N., Udagawa Y., Ito M. (1972). Resonance Raman effect of I_3_^−^ ion by ultraviolet laser excitation. Chem. Phys. Lett..

[44] Gabes W., Stufkens D.J. (1974). Electronic absorption spectra of symmetrical and asymmetrical trihalide ions. Spectrochim. Acta Part A.

[45] Stappen C.V., Lehnert N. (2018). Mechanism of N-N bond formation by transition metal-nitrosyl complexes: Modeling flavodiiron nitric oxide reductases. Inorg. Chem..

[46] Armarego W.L.F., Chai C.L.L. (2012). Purification of Laboratory Chemicals.

[47] Frisch M.J., Trucks G.W., Schlegel H.B., Scuseria G.E., Robb M.A., Cheeseman J.R., Scalmani G., Barone V., Petersson G.A., Nakatsuji H. (2016). Gaussian 16, Revision C.01.

[48] Schäfer A., Horn H., Ahlrichs R. (1992). Fully optimized contracted Gaussian basis sets for atoms Li to Kr. J. Chem. Phys..

[49] Weigend F., Ahlrichs R. (2005). Balanced basis sets of split valence, triple zeta valence and quadruple zeta valence quality for H to Rn: Design and assessment of accuracy. Phys. Chem. Chem. Phys..

[50] Becke A.D. (1988). Density-functional exchange-energy approximation with correct asymptotic behavior. Phys. Rev. A.

[51] Perdew J.P. (1986). Density-functional approximation for the correlation energy of the inhomogeneous electron gas. Phys. Rev. B.

[52] Becke A.D. (1993). Density-functional thermochemistry. III. the role of exact exchange. J. Chem. Phys..

[53] Lee C., Yang W., Parr R.G. (1988). Development of the Colle-Salvetti correlation-energy formula into a functional of the electron density. Phys. Rev. B.

[54] (2001). CrystalClear.

[55] (2015). CrysAlisPro.

[56] Burla M.C., Caliandro R., Camalli M., Carrozzini B., Cascarano G.L., De Caro L., Giacovazzo C., Polidori G., Siliqi D., Spagna R. (2007). *IL MILIONE*: A suite of computer programs for crystal structure solution of proteins. J. Appl. Crystallogr..

[57] (2003). Crystal Structure.

[58] Sheldrick G.M. (2015). Crystal Structure Refinement with *SHELXL*. Acta Crystallogr..

