# Supplementary material for: Coordinatively Unsaturated Nickel Nitroxyl Complex: Structure, Physicochemical Properties, and Reactivity toward Dioxygen
Source: Molecules. 2023 Aug 23;28(17):6206. doi: 10.3390/molecules28176206 (PMC10489029; doi:10.3390/molecules28176206)
Supplement: Supplementary file 1 [file molecules-28-06206-s001.zip › molecules-2564965-supplementary.pdf]

Supporting information

Coordinatively Unsaturated Nickel Nitrosyl Complex: Structure, Physicochemical Properties, and Reactivity toward Dioxygen

Kiyoshi Fujisawa, Taisei Kataoka, Kohei Terashima,
Felipe de Santis Gonçalves, Nicolai Lehnert

Content

Figure S1. IR spectrum of **[Ni(NO)(I)(L1″)]** (KBr). S2

Figure S2. Far-IR spectrum of **[Ni(NO)(I)(L1″)]** (KBr). S2

Figure S3. IR and UV-Vis spectra of the starting material [Ni(NO)(I)]*n*. S3

Figure S4. UV–Vis spectrum of **[Ni(NO)(I)(L1″)]** in solid state (nujol). S3

Figure S5. ^{1}H-NMR spectrum of **[Ni(NO)(I)(L1″)]** in $CDCl_3$-d_1. S4

Figure S6. ^{1}H-NMR spectrum of **[Ni(NO)(I)(L1″)]** in $(CD_3)_2CO$-d_6. S4

Figure S7. ^{13}C-NMR spectrum of **[Ni(NO)(I)(L1″)]** in $CDCl_3$-d_1. S5

Figure S8. IR spectrum of **[Ni(κ^2-NO$_2$)(L1″)$_2$](I$_3$)** (KBr). S6

Figure S9. Far-IR spectrum of **[Ni(κ^2-NO$_2$)(L1″)$_2$](I$_3$)** (KBr). S6

Figure S10. IR spectral comparisons of the reaction of **[Ni(NO)(I)(L1″)]** with $^{16}O_2$ and $^{18}O_2$. S7

Figure S11. UV-Vis spectrum of **[Ni(κ^2-O$_2$N)(L1″)$_2$](I$_3$)** in solid state (nujol). S7

Table S1. XYZ coordinates for the optimized structure of **[Ni(NO)(I)(L1″)]** using the BP86 functional for the closed-shell system. S8

Table S2. XYZ coordinates for the optimized structure of **[Ni(NO)(I)(L1″)]** using the B3LYP functional for the closed-shell system. S10

Table S3. XYZ coordinates for the optimized structure of **[Ni(NO)(I)(L1″)]** using the B3LYP functional for the broken-symmetry system. S12

Table S4. XYZ coordinates for the optimized structure of **[Ni(κ^2-O$_2$N)(L1″)$_2$]** using the BP86 functional. S14

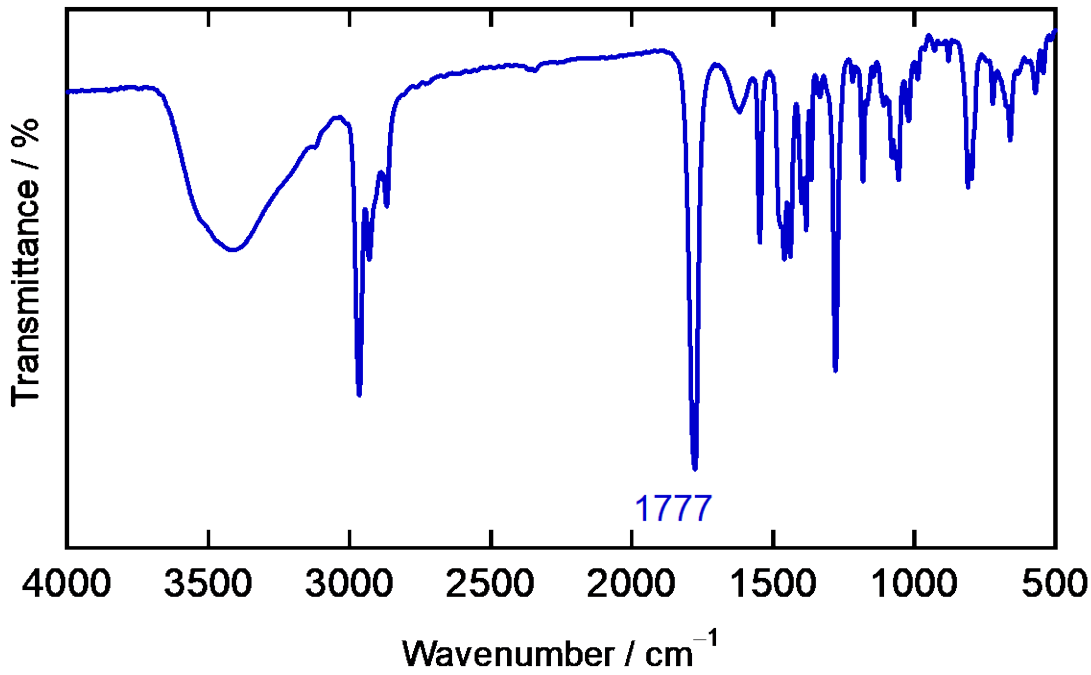

Figure S1. IR spectrum of **[Ni(NO)(I)(L1″)]** (KBr) at room temperature.

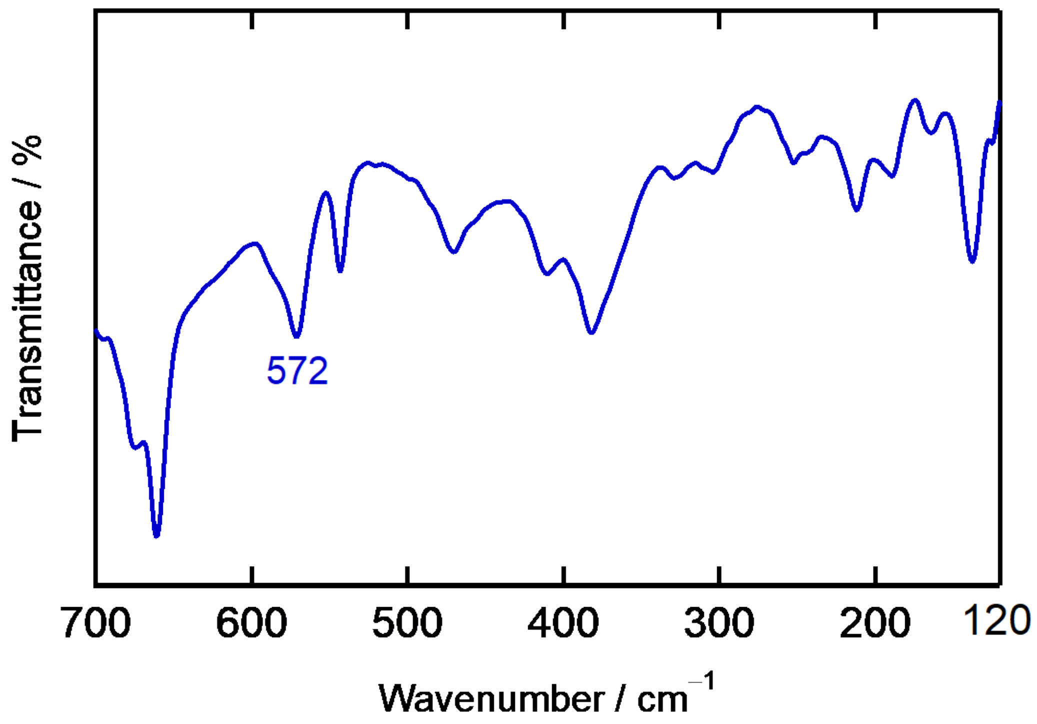

Figure S2. Far-IR spectrum of **[Ni(NO)(I)(L1″)]** (CsI) at room temperature.

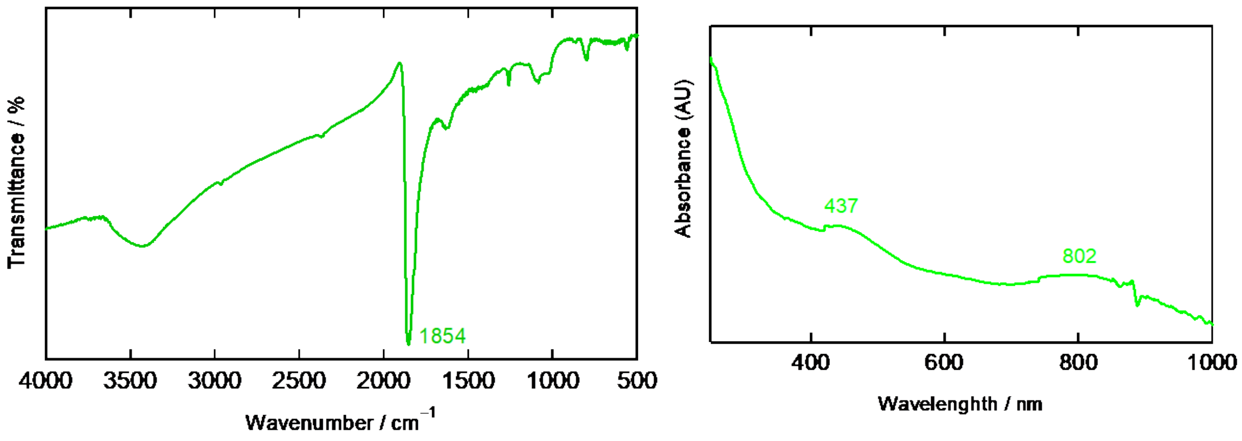

Figure S3. IR (KBr, left) and UV-Vis (nujol, right) spectra of the starting material [Ni(NO)(I)]*n*, obtained at room temperature.

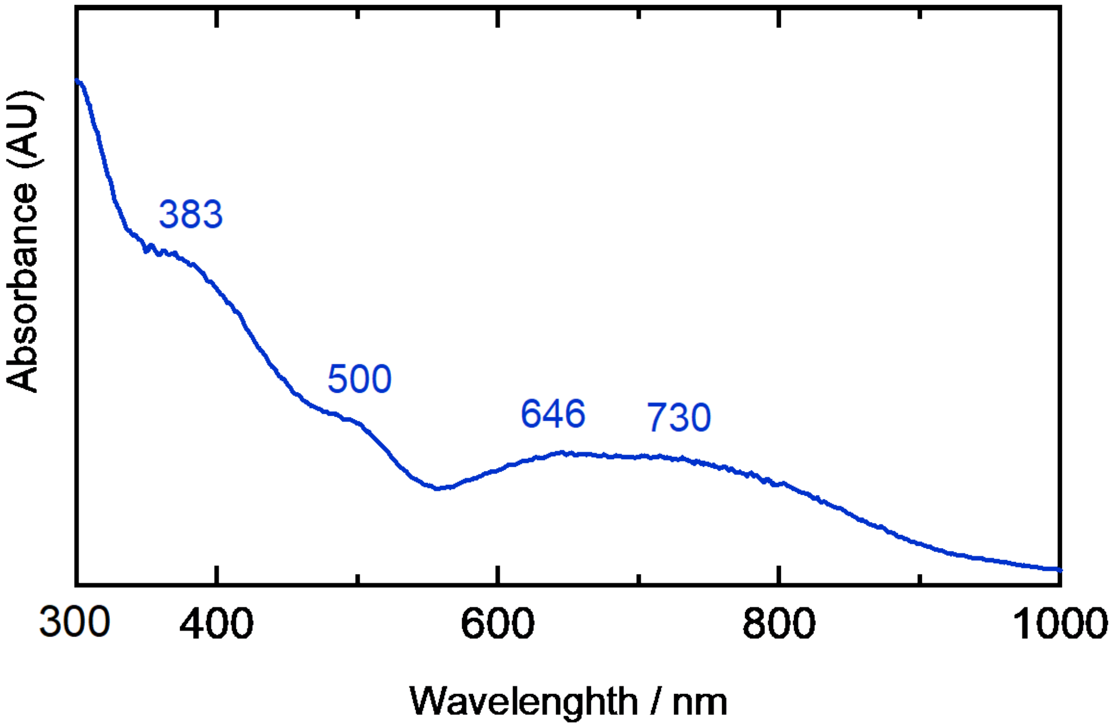

Figure S4. UV–Vis spectrum of **[Ni(NO)(I)(L1'')]** in the solid state (nujol) at room temperature.

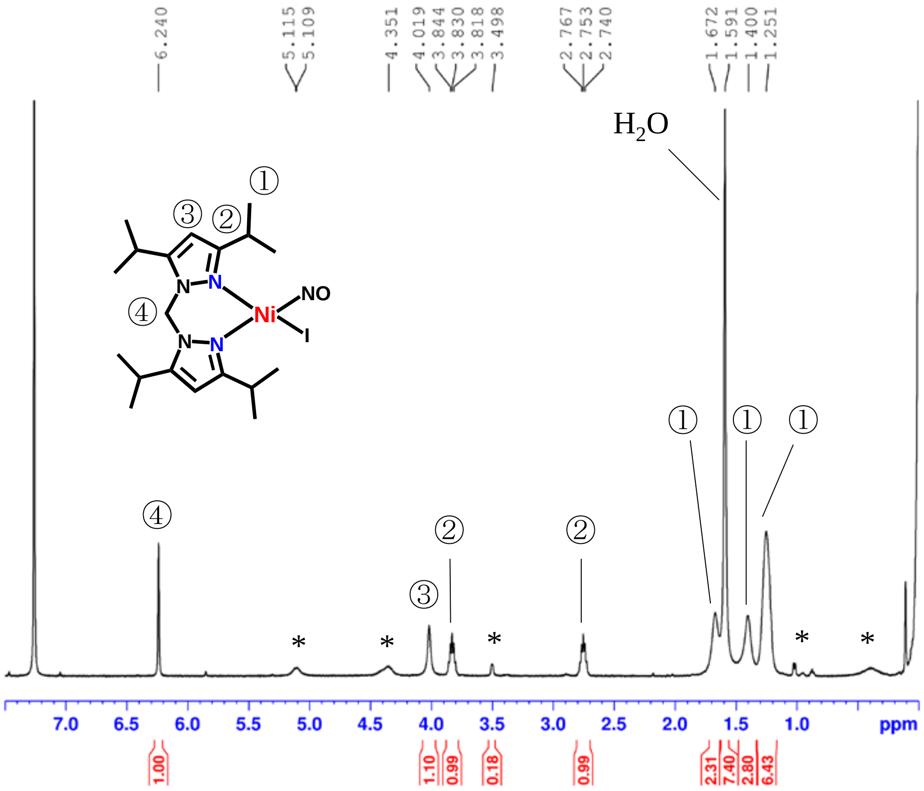

Figure S5. ^{1}H-NMR spectrum of **[Ni(NO)(I)(L1′′)]** in $CDCl_3$-*d*[1] at room temperature (* marks solvents and impurities peaks).

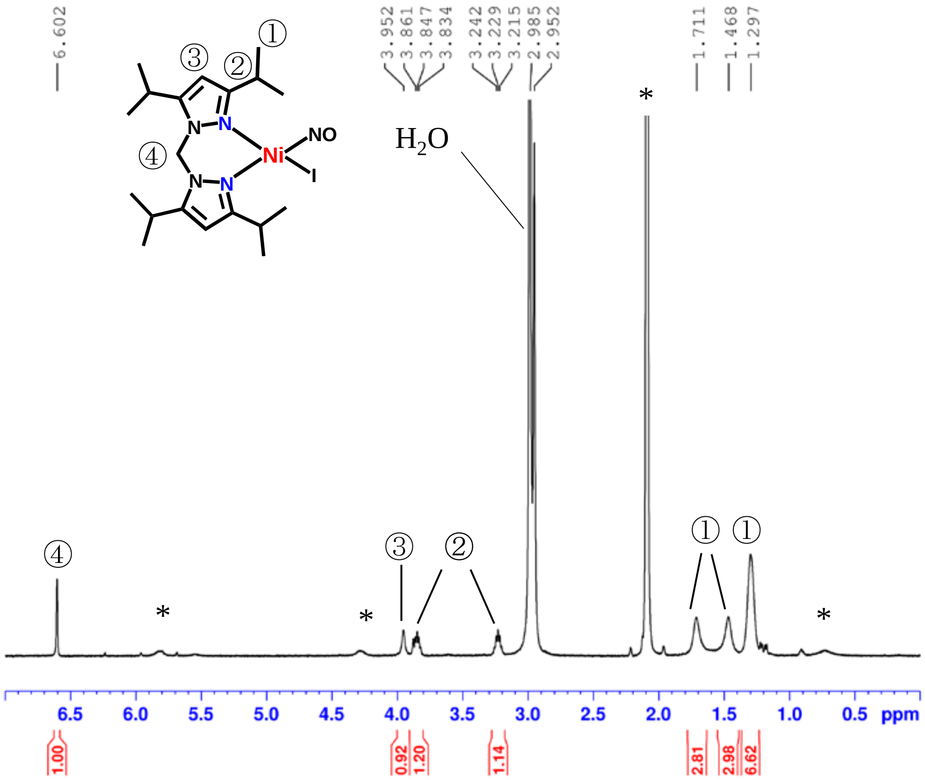

Figure S6. ^{1}H-NMR spectrum of **[Ni(NO)(I)(L1′′)]** in $(CD_3)_2CO$-*d*[6] at room temperature (* marks solvents and impurities peaks).

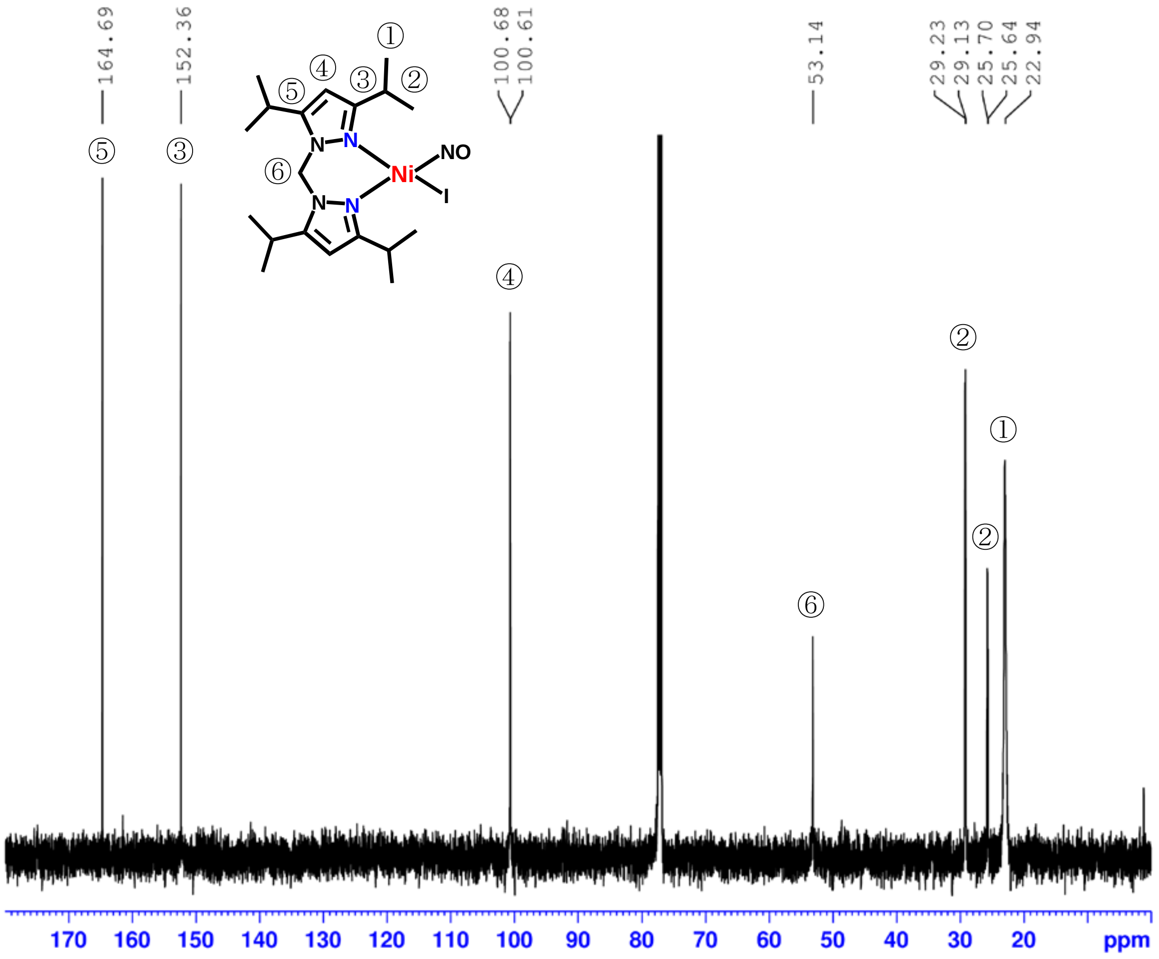

Figure S7. 13C-NMR spectrum of **[Ni(NO)(I)(L1′′)]** in $CDCl_3$-d_1 at room temperature (* marks solvents peaks).

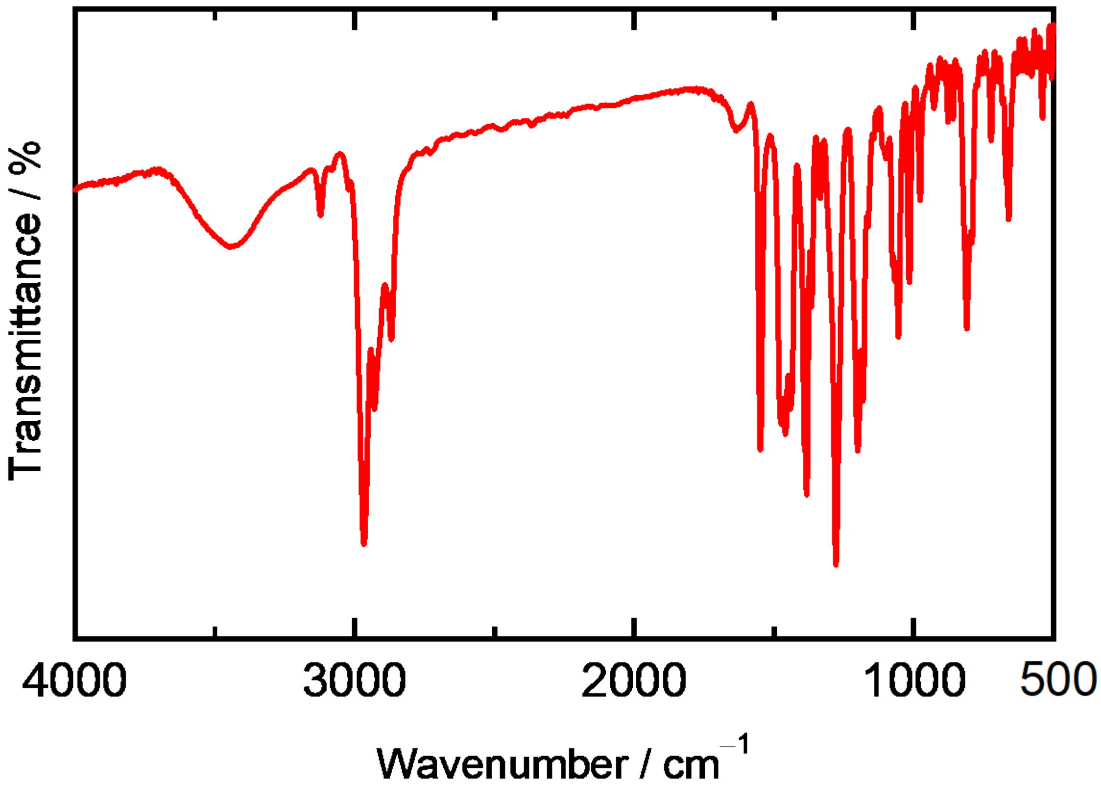

Figure S8. IR spectrum of **[Ni(κ²-NO₂)(L1′′)₂](I₃)** (KBr) at room temperature.

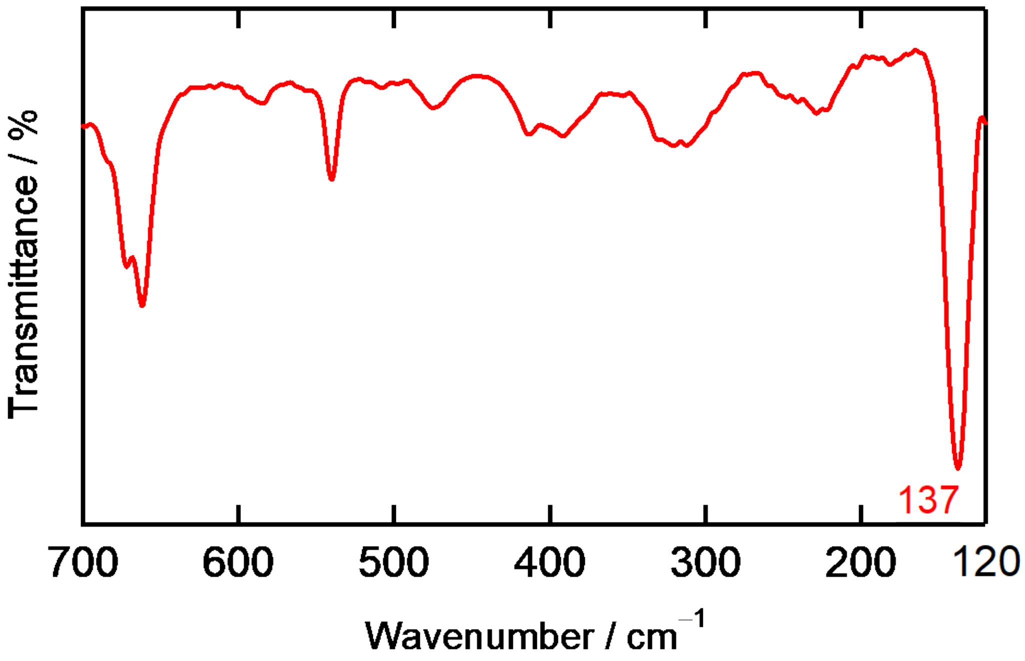

Figure S9. Far-IR spectrum of **[Ni(κ²-NO₂)(L1′′)₂](I₃)** (CsI) at room temperature.

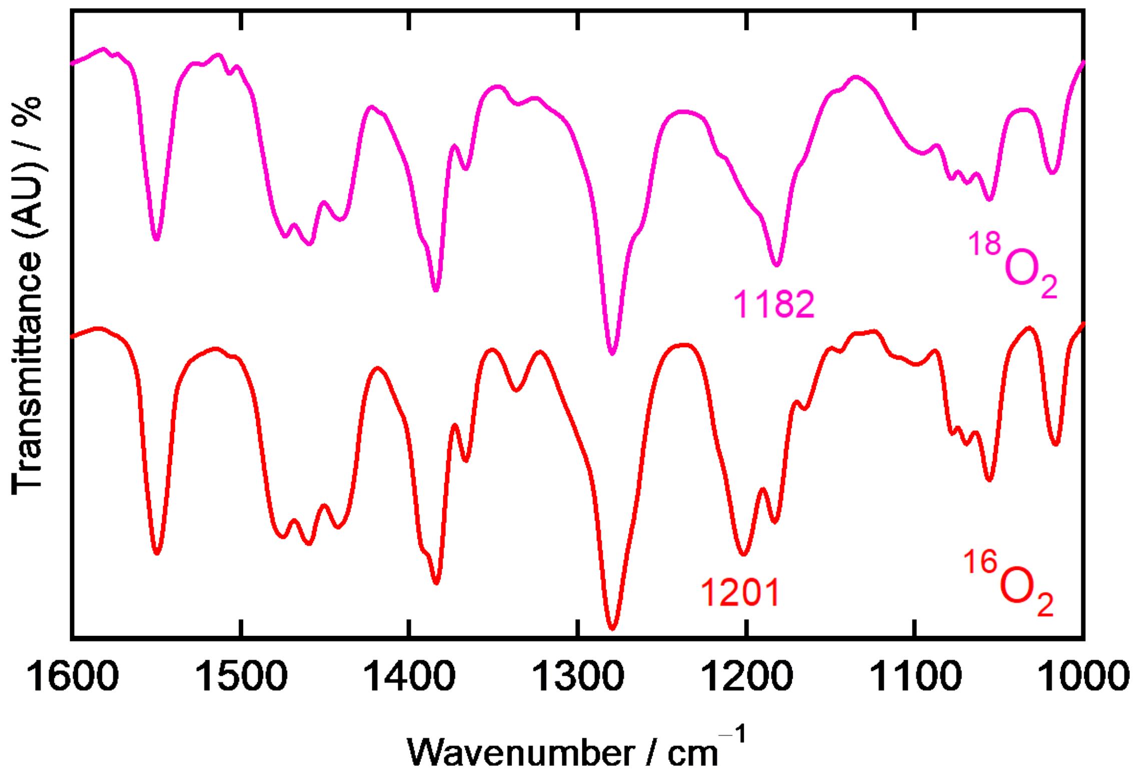

Figure S10. IR spectral comparisons of the products obtained from the reaction of **[Ni(NO)(I)(L1″)]** with $^{16}O_2$ (bottom) and $^{18}O_2$ (top) (KBr).

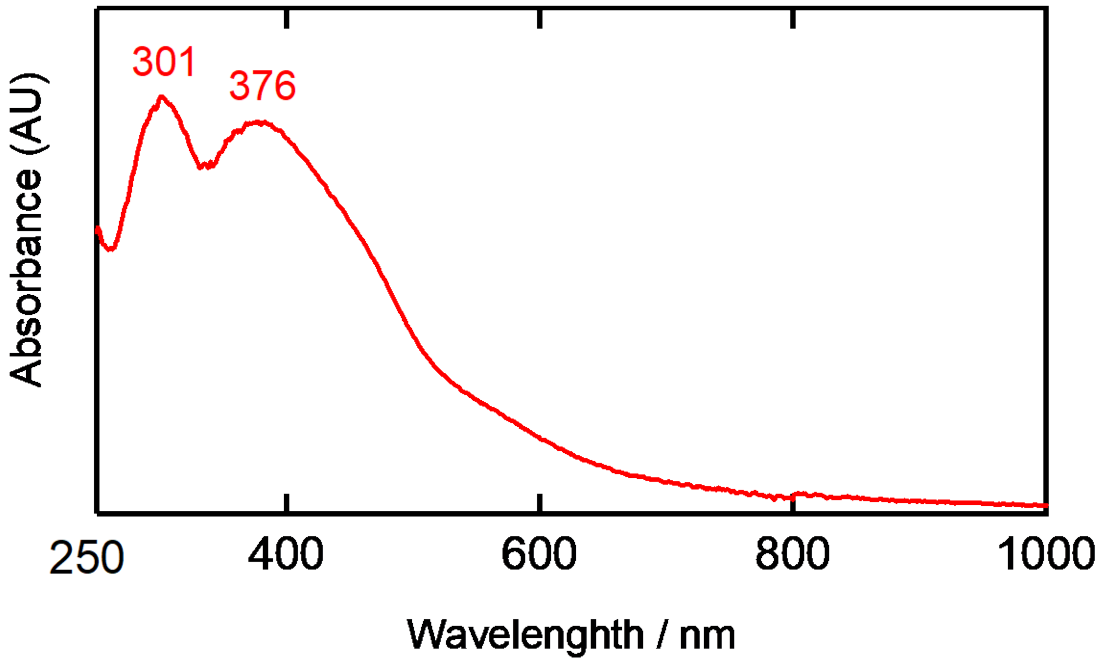

Figure S11. UV-Vis spectrum of **[Ni(κ²-O₂N)(L1″)₂](I₃)** in the solid state (nujol) at room temperature.

Table S1. XYZ coordinates for the optimized structure of **[Ni(NO)(I)(L1′′)]** using the BP86 functional for the closed-shell system.

	x	y	z
I	-0.0069	-1.93962	2.2655
Ni	-0.00424	-1.45249	-0.3308
O	-0.01192	-4.12034	-1.16455
N	-0.00858	-2.96171	-0.9884
N	1.42264	-0.02659	-0.45349
N	1.2074	1.22552	0.05015
N	-1.42227	-0.01791	-0.45408
N	-1.19956	1.23287	0.04961
C	4.04618	-0.86909	-2.94939
H	4.40322	-1.79056	-3.43226
H	3.46637	-0.29785	-3.68923
H	4.93401	-0.27578	-2.67907
C	4.01726	-2.03037	-0.68547
H	4.38872	-2.95886	-1.1452
H	4.88591	-1.45542	-0.32786
H	3.40048	-2.29753	0.18396
C	3.20464	-1.21257	-1.71115
H	2.34879	-1.83195	-2.03027
C	2.62057	0.01506	-1.06519
C	3.16972	1.31204	-0.94158
H	4.12044	1.65629	-1.33383
C	2.25143	2.07094	-0.22504
C	2.31399	3.49668	0.25745
H	1.28808	3.9089	0.23428
C	3.17481	4.36501	-0.67355
H	3.16299	5.41109	-0.33452
H	4.22242	4.0282	-0.66993
H	2.80888	4.33261	-1.70945
C	2.82546	3.57623	1.71265
H	2.81583	4.61794	2.06757
H	2.21288	2.97452	2.39968
H	3.85769	3.20096	1.77562
C	-4.02799	-2.00651	-0.68596
H	-4.40512	-2.9327	-1.14572
H	-3.41226	-2.27749	0.18302
H	-4.89306	-1.42662	-0.32766
C	-4.05152	-0.84457	-2.94959

H	-4.41419	-1.76385	-3.43241
H	-4.93574	-0.24618	-2.6786
H	-3.46887	-0.27654	-3.68967
C	-3.21126	-1.19316	-1.71191
H	-2.35916	-1.81738	-2.03164
C	-2.61982	0.03096	-1.06597
C	-3.16115	1.33124	-0.94246
H	-4.1097	1.68122	-1.33491
C	-2.23842	2.08458	-0.22576
C	-2.29239	3.51069	0.25668
H	-1.26399	3.91668	0.23353
C	-3.1479	4.38417	-0.67439
H	-3.12978	5.43016	-0.3354
H	-2.78213	4.34951	-1.71027
H	-4.19752	4.05367	-0.67079
C	-2.80343	3.59341	1.71186
H	-2.78741	4.63504	2.06676
H	-3.83795	3.2245	1.77479
H	-2.1946	2.98795	2.39893
C	0.00439	1.44078	0.83361
H	0.00741	2.46508	1.21686
H	0.00196	0.7082	1.66533

Table S2. XYZ coordinates for the optimized structure of **[Ni(NO)(I)(L1'')]** using the B3LYP functional for the closed-shell system.

	x	y	z
I	-0.01144	-2.01811	2.25943
Ni	-0.0075	-1.48867	-0.34956
O	-0.02014	-4.1182	-1.18563
N	-0.01469	-2.97396	-1.02121
N	1.43765	-0.01102	-0.46159
N	1.20877	1.22476	0.05333
N	-1.43725	0.00371	-0.46217
N	-1.19576	1.23716	0.05258
C	3.95242	-0.81059	-3.01799
H	4.32475	-1.71634	-3.49969
H	3.28691	-0.30326	-3.71856
H	4.81135	-0.16017	-2.83609
C	4.16476	-1.9033	-0.73825
H	4.55593	-2.81079	-1.20283
H	5.01344	-1.27406	-0.45921
H	3.63609	-2.18602	0.17232
C	3.23147	-1.1657	-1.71305
H	2.40297	-1.83692	-1.95159
C	2.62687	0.04243	-1.05574
C	3.16842	1.33516	-0.91275
H	4.11308	1.68582	-1.28858
C	2.24541	2.07155	-0.19869
C	2.30302	3.48909	0.30031
H	1.28806	3.89993	0.27006
C	3.17183	4.36847	-0.60516
H	3.15184	5.40148	-0.25449
H	4.21208	4.03786	-0.59452
H	2.8205	4.35044	-1.6374
C	2.79513	3.54934	1.75802
H	2.78021	4.57844	2.12243
H	2.17853	2.9445	2.42461
H	3.81856	3.17695	1.82973
C	-4.18056	-1.8625	-0.73699
H	-4.58096	-2.76613	-1.2012
H	-3.65254	-2.15049	0.17232
H	-5.02286	-1.22568	-0.45584
C	-3.96311	-0.77047	-3.01658

H	-4.34461	-1.67253	-3.49803
H	-4.81577	-0.11248	-2.83245
H	-3.29455	-0.26872	-3.71827
C	-3.24267	-1.13288	-1.7134
H	-2.42083	-1.81151	-1.95406
C	-2.62572	0.06928	-1.05659
C	-3.15393	1.36758	-0.91405
H	-4.09478	1.7279	-1.29026
C	-2.22354	2.09453	-0.1999
C	-2.26657	3.51273	0.29868
H	-1.24735	3.91296	0.26868
C	-3.12582	4.40086	-0.60737
H	-3.09523	5.43371	-0.25699
H	-2.77432	4.37885	-1.63947
H	-4.16946	4.08106	-0.59701
C	-2.75855	3.57856	1.75619
H	-2.73293	4.60755	2.12033
H	-3.78586	3.217	1.82763
H	-2.14859	2.96746	2.42318
C	0.00728	1.42866	0.83483
H	0.01235	2.43737	1.22887
H	0.00329	0.70269	1.65356

Table S3. XYZ coordinates for the optimized structure of **[Ni(NO)(I)(L1′′)]** using the B3LYP functional for the broken-symmetry system.

	x	y	z
I	-0.15856	-1.98903	2.30843
Ni	-0.11274	-1.48798	-0.30096
O	-0.32003	-4.12078	-1.42972
N	-0.22962	-2.9974	-1.11829
N	1.44023	-0.1232	-0.43938
N	1.2946	1.13567	0.05043
N	-1.43748	0.09681	-0.44611
N	-1.10381	1.31942	0.04314
C	3.74115	-1.15505	-3.05453
H	4.06085	-2.09225	-3.51344
H	3.0072	-0.68817	-3.71354
H	4.61367	-0.49944	-3.00243
C	4.17916	-2.11076	-0.74466
H	4.51336	-3.04957	-1.19069
H	5.05738	-1.47886	-0.59136
H	3.74421	-2.33269	0.23022
C	3.15616	-1.42123	-1.66217
H	2.30294	-2.09394	-1.7732
C	2.63341	-0.16498	-1.02576
C	3.26353	1.08917	-0.90287
H	4.23343	1.36484	-1.27711
C	2.39062	1.90164	-0.20904
C	2.54673	3.31983	0.26651
H	1.5634	3.8006	0.22572
C	3.47803	4.12013	-0.65013
H	3.53029	5.15766	-0.3167
H	4.4922	3.71694	-0.62986
H	3.12915	4.10967	-1.6833
C	3.03813	3.36903	1.72489
H	3.09549	4.40254	2.07206
H	2.37838	2.82089	2.39917
H	4.03229	2.92616	1.80655
C	-4.34675	-1.53735	-0.68708
H	-4.817	-2.418	-1.12911
H	-3.86479	-1.83878	0.24329
H	-5.13393	-0.81838	-0.44728
C	-3.9859	-0.55416	-2.9985

H	-4.43799	-1.43562	-3.45628
H	-4.77817	0.18503	-2.85789
H	-3.26014	-0.14189	-3.7016
C	-3.32773	-0.93103	-1.66654
H	-2.56643	-1.68924	-1.86394
C	-2.62066	0.23621	-1.03803
C	-3.05203	1.57212	-0.92038
H	-3.96586	1.99235	-1.30107
C	-2.06818	2.24336	-0.22348
C	-2.00631	3.67063	0.24634
H	-0.96021	3.99331	0.2104
C	-2.79693	4.60123	-0.67939
H	-2.69	5.63587	-0.35002
H	-2.44754	4.53215	-1.71012
H	-3.86137	4.35991	-0.66456
C	-2.49299	3.80196	1.70122
H	-2.39092	4.8333	2.0446
H	-3.54444	3.51961	1.77777
H	-1.9307	3.16071	2.38167
C	0.10906	1.4362	0.82556
H	0.18531	2.44778	1.20444
H	0.05181	0.72483	1.65536

Table S4. XYZ coordinates for the optimized structure of **[Ni(κ²-O₂N)(L1′′)₂]** using the BP86 functional.

	x	y	z
Ni	-0.04748	0.08459	-0.79287
O	0.39703	0.89362	-2.68722
O	-0.66772	-0.92188	-2.62159
N	-0.15382	-0.03331	-3.3666
N	-0.69069	-1.10983	0.84231
N	-2.00033	-1.52401	0.83014
N	-2.02062	0.90739	-0.97047
N	-3.0645	0.06677	-0.66508
N	1.7569	-1.02714	-1.03329
N	2.95211	-0.36436	-0.93267
N	0.90538	1.57128	0.40069
N	2.28076	1.60039	0.33116
C	2.09711	-2.56824	2.37328
H	3.10982	-2.36876	2.75414
H	1.66773	-3.37997	2.98016
H	2.18781	-2.92042	1.33872
C	1.28103	-0.75488	3.91435
H	2.32086	-0.54264	4.20228
H	0.69891	0.16735	4.03101
H	0.89061	-1.49818	4.62605
C	1.23836	-1.29179	2.47064
H	1.66862	-0.53389	1.80033
C	-0.18046	-1.54178	2.01686
C	-1.17268	-2.24795	2.73326
H	-1.0607	-2.71572	3.7053
C	-2.32553	-2.22833	1.96218
C	-3.69529	-2.78623	2.25295
H	-4.16892	-3.06391	1.29439
C	-3.60954	-4.06529	3.10209
H	-4.61449	-4.48412	3.25226
H	-2.98629	-4.83136	2.62035
H	-3.18878	-3.854	4.09631
C	-4.58801	-1.72788	2.93711
H	-5.60128	-2.12593	3.09274
H	-4.17434	-1.45394	3.91878
H	-4.66733	-0.80775	2.33977
C	-1.82614	3.26296	-3.50658
H	-1.21893	4.10724	-3.86527

H	-1.43295	2.34403	-3.95835
H	-2.85581	3.41351	-3.86545
C	-2.31328	4.53415	-1.38116
H	-1.67091	5.36741	-1.70137
H	-3.33084	4.74906	-1.7415
H	-2.3405	4.52363	-0.28361
C	-1.7897	3.20857	-1.96403
H	-0.74511	3.06062	-1.64737
C	-2.58349	2.0311	-1.459
C	-3.99122	1.89771	-1.45806
H	-4.71236	2.64097	-1.78012
C	-4.27882	0.63447	-0.96578
C	-5.59988	-0.07653	-0.81851
H	-5.52336	-0.77013	0.03815
C	-5.92879	-0.90521	-2.08039
H	-6.87301	-1.45159	-1.94137
H	-6.03995	-0.24613	-2.95367
H	-5.14287	-1.63786	-2.31665
C	-6.73383	0.9129	-0.50326
H	-7.67497	0.36793	-0.34436
H	-6.52007	1.50219	0.39936
H	-6.89422	1.61016	-1.3388
C	-2.78114	-1.32773	-0.3765
H	-2.23779	-1.75974	-1.22954
H	-3.73486	-1.84877	-0.25422
C	1.23483	-4.64042	-0.79256
H	0.48107	-5.39223	-1.06918
H	2.22431	-5.08592	-0.97633
H	1.14204	-4.45008	0.28496
C	1.03907	-3.70913	-3.13457
H	0.24849	-4.44068	-3.35798
H	0.86664	-2.81967	-3.75362
H	2.00158	-4.15586	-3.42713
C	1.02937	-3.36489	-1.62959
H	0.04703	-2.93585	-1.38262
C	2.06324	-2.30356	-1.34203
C	3.46805	-2.44235	-1.43467
H	4.0158	-3.35037	-1.66266
C	4.01745	-1.19309	-1.1879
C	5.45026	-0.72786	-1.24228

H	5.57393	0.0898	-0.50942
C	5.80211	-0.17541	-2.64173
H	6.83598	0.19919	-2.65396
H	5.71606	-0.96767	-3.39957
H	5.13856	0.64772	-2.9457
C	6.41828	-1.85232	-0.8396
H	7.45054	-1.47509	-0.83184
H	6.1887	-2.24885	0.15925
H	6.37767	-2.68498	-1.55734
C	-0.99494	4.15351	2.12776
H	-2.0517	4.40355	2.3018
H	-0.43565	4.46035	3.02475
H	-0.6321	4.75421	1.28251
C	-1.3215	1.85455	3.105
H	-2.35757	2.13563	3.34587
H	-1.29262	0.76995	2.93776
H	-0.70344	2.08717	3.98593
C	-0.84388	2.64086	1.87028
H	-1.48296	2.35872	1.02073
C	0.5775	2.32556	1.47202
C	1.75054	2.82953	2.07747
H	1.80456	3.483	2.94111
C	2.82464	2.36738	1.33289
C	4.30308	2.59164	1.52092
H	4.77608	2.63809	0.52331
C	4.94802	1.42313	2.29768
H	6.0347	1.57168	2.37762
H	4.53653	1.36757	3.31629
H	4.76834	0.45442	1.80886
C	4.5826	3.93362	2.21672
H	5.66651	4.10101	2.28648
H	4.13979	4.77594	1.66741
H	4.18172	3.94056	3.24119
C	2.92957	1.08588	-0.86131
H	2.39882	1.47504	-1.74256
H	3.96385	1.44168	-0.87032
